# An Integrated Mathematical Model of Thrombin-, Histamine-and VEGF-Mediated Signalling in Endothelial Permeability

**DOI:** 10.1186/1752-0509-5-112

**Published:** 2011-07-15

**Authors:** XN Wei, BC Han, JX Zhang, XH Liu, CY Tan, YY Jiang, BC Low, B Tidor, YZ Chen

**Affiliations:** 1 Computation and Systems Biology, Singapore-MIT Alliance, National University of Singapore, E4-04-10, 4 Engineering Drive 3, 117576, Singapore; 2 The Guangdong Provincial Key Laboratory of Chemical Biology, The Graduate School at Shenzhen, Tsinghua University, Shenzhen, China; 3 Bioinformatics and Drug Design Group, Department of Pharmacy and Center of Computational Science and Engineering, National University of Singapore, Blk S16, Level 8, 3 Science Drive, 117543, Singapore; 4 Research Centre of Excellence in Mechanobiology Institute, Cell Signalling and Developmental Biology Laboratory, Department of Biological Sciences, National University of Singapore, 14 Science Drive 4, 117543, Singapore; 5 Department of Biological Engineering, Department of Electrical Engineering & Computer Science, Computer Science and Artificial Intelligence Laboratory, Massachusetts Institute of Technology, Room 32-212, Cambridge, MA 02139-4307 USA; 6 Singapore-MIT Alliance for Research and Technology, Blk S16, Level 7, 3 Science Drive 2, 117543, Singapore

## Abstract

**Background:**

Endothelial permeability is involved in injury, inflammation, diabetes and cancer. It is partly regulated by the thrombin-, histamine-, and VEGF-mediated myosin-light-chain (MLC) activation pathways. While these pathways have been investigated, questions such as temporal effects and the dynamics of multi-mediator regulation remain to be fully studied. Mathematical modeling of these pathways facilitates such studies. Based on the published ordinary differential equation models of the pathway components, we developed an integrated model of thrombin-, histamine-, and VEGF-mediated MLC activation pathways.

**Results:**

Our model was validated against experimental data for calcium release and thrombin-, histamine-, and VEGF-mediated MLC activation. The simulated effects of PAR-1, Rho GTPase, ROCK, VEGF and VEGFR2 over-expression on MLC activation, and the collective modulation by thrombin and histamine are consistent with experimental findings. Our model was used to predict enhanced MLC activation by CPI-17 over-expression and by synergistic action of thrombin and VEGF at low mediator levels. These may have impact in endothelial permeability and metastasis in cancer patients with blood coagulation.

**Conclusion:**

Our model was validated against a number of experimental findings and the observed synergistic effects of low concentrations of thrombin and histamine in mediating the activation of MLC. It can be used to predict the effects of altered pathway components, collective actions of multiple mediators and the potential impact to various diseases. Similar to the published models of other pathways, our model can potentially be used to identify important disease genes through sensitivity analysis of signalling components.

## Background

The endothelium is a semi-permeable barrier that regulates the flux of liquid and solutes between the blood and surrounding tissues. Endothelial permeability increases paracellular leakage of plasma fluid and proteins to surrounding tissues, and intravasation of tissue-released contents to the blood in the development of multiple diseases related to injury (such as edema, trauma, ischaemia-reperfusion injury, respiratory distress syndrome, and thrombosis), inflammation (such as atherosclerosis and sepsis), diabetes, and cancer [1-4]. The level of endothelial permeability is regulated individually or in combination by multiple mediators, particularly thrombin, histamine, and vascular endothelial growth factor (VEGF), under various disease conditions [4].

The proinflammatory and vasoactive factors thrombin, generated in thrombosis and inflammatory diseases, and histamine, produced in acute inflammatory responses to trauma, burns, allergy, and infection, induce transient endothelial permeability to link inflammation, tissue injury and vascular leakage to cellular responses and symptoms [5-7]. VEGF, released in diabetic retinopathy, I-R injury, vasculogenesis, angiogenesis, and tumor development and metastasis, causes endothelial permeability to enable extravasation of fluids and solutes and intravasation of tumor cells [8-10]. These three key mediators stimulate their respective receptors on endothelial cells to individually and collectively activate Ca^2+^, Rho GTPase/ROCK, and Myosin light chain kinase (MLCK) signalling pathways that subsequently activate myosin light chain (MLC) to induce cytoskeleton contraction in endothelial cells and dissociation of cell-cell junctions, resulting in endothelial hyper-permeability [4,11].

Significant progress has been made in understanding the molecular mechanism and dynamics of the relevant signalling events [4,7,9,11,12] and the roles of different regulators [13,14]. Nonetheless, some puzzles still remain to be elucidated. For instance, it is unclear what contributes to the different temporal effects and permeability recovery rates by histamine, thrombin, and VEGF mediated signalling, given that they share similar signalling cascades in triggering endothelial permeability. Another question is how multiple mediators under certain complicated inflammatory conditions collectively reduce the effectiveness of antagonizing agents directed at individual mediator-mediated signalling [4].

As part of the efforts for solving these puzzles and for quantitative and mechanistic study of the relevant signalling events, mathematical models have been developed for analyzing the relevant signalling and regulation processes [15-20]. In particular, ordinary differential equation (ODE) based mathematical models of thrombin, Ca^2+^-calmodulin (CaM), and Rho activation have been developed for investigating the thrombin-mediated activation of MLC [18], and Ca^2+^-CaM, MLCK and Myosin Light chain phosphatase (MYCP) on MLC activation [15,16,21]. To enable more comprehensive analysis of signalling in endothelial permeability, there is a need to develop an expanded ODE model that covers the signalling mediated by multiple mediators, particularly thrombin, histamine and VEGF.

**Figure 1:**
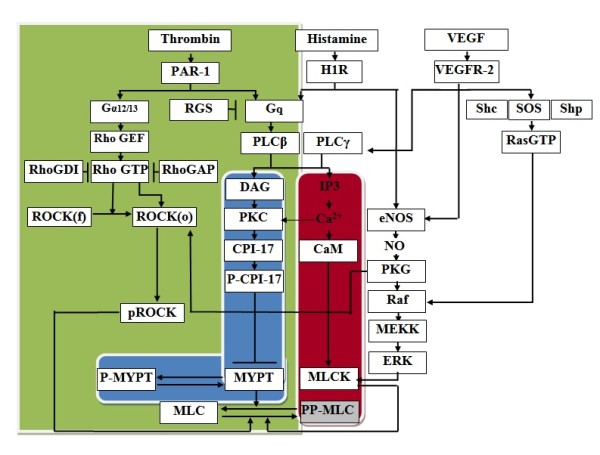
**Framework of integrated pathway simulation model of thrombin-, histamine-, and VEGF-mediated MLC activation**. The components in existing models are highlighted by red, blue, and red + blue + green background colour for models from reference 15, 16 and 18 respectively. The protein in the gray box represents output signalling. ROCK (f) and ROCK (o) refer to ROCK in folded and open conformation respectively.

In this work, we developed a mathematical model that integrates thrombin, histamine, and VEGF mediated signalling in endothelial permeability by extending the published ODE models of the thrombin-mediated pathway and Ca^2+^-CaM and MLCK activation of MLC [15,16,18,21]. The framework of our integrated mathematical model is illustrated in Figure 1 and the detailed pathway maps of all three signalling components and thrombin-, histamine- and VEGF-mediated signalling cascades are given in Additional File 1, Figure S1, S2 and S3 respectively. Detailed molecular interactions and the corresponding kinetic data were obtained from the literature, including published simulation models [15,16,18,21], which are summarized in Additional File 2. Our model was validated by evaluating whether the time course of MLC activation by each individual mediator (thrombin, histamine, and VEGF) is in agreement with published experimental and computational findings. The sensitivity of our model with respect to parameters was analyzed to evaluate its robustness. The validated model was then used to study the modulation of other pathway components by each individual mediator (thrombin, histamine, and VEGF) [4,11] and the modulation of MLC activation by combination of a pair of key mediators thrombin and histamine [22,23]. Our model was further used to predict the regulation of MLC activation by PKC-potentiated inhibitory protein of 17 kDa (CPI-17) over-expression and by combination of thrombin and VEGF at low mediator levels. The effects of the protein variation of key signalling components protease-activated receptor-1 (PAR-1), VEGF, VEGFR2, Rho GTPase, and ROCK on MLC activation were also studied. Some of these components are significantly elevated in different diseases and have been explored as therapeutic targets for pharmacological intervention of endothelial permeability and barrier function in these diseases [20].

## Thrombin-, Histamine-and VEGF-Mediated Signalling Cascades in Endothelial Permeability

### Thrombin mediated GPCR activation

Thrombin regulates endothelial permeability, inflammation and other events via activation of thrombin receptors such as PAR-1 by proteolytically cleaving the N-terminus of these receptors [24]. PAR-1 is the main receptor in the regulation of endothelial permeability (Additional File 1, Figure S1). It interacts with Gq to increase the concentration of Ca^2+ ^and activate protein kinase C, inositol 1, 4, 5-triphosphate and diacylglycerol [25]. It is also linked to G12/13 [26] to activate the small G-protein Rho [27].

### VEGF mediated ERK activation

VEGF regulates angiogenesis, cancer and microvascular permeability under various physiological and pathological conditions by activating transmembrane tyrosine kinase receptors VEGFR-2 and Flt-1, which promotes mitogenic, chemotactic, and prosurvival signalling and activates phospholipase C (PLC), intracellular Ca^2+^, and various protein kinase C (PKC) isoforms. In particular, VEGF activates ERK-1/2 via the Raf-MEK-ERK cascade [28]. Accumulative evidences suggest that ERK-MLCK-meditated cytoskeletal responses contribute to VEGF-elicited microvascular hyperpermeability. For instance, MLCK has been found to contain multiple MAP kinase consensus phosphorylation sites (P-x-S[29]-P) that can be directly phosphorylated by MAP kinase [30], which is supported by additional experimental evidence indicating MLCK as a substrate for MAP kinase [31].

### Histamine, VEGF mediated NO activation

Cytosolic Ca^2+ ^elevation is a typical initial response of endothelial cells to hormonal and chemical signal and to changes in physical parameters, and many endothelial functions are dependent on changes in Ca^2+ ^concentration [32]. For instance, the activity of endothelial nitric oxide synthase (eNOS) in producing nitric oxide in endothelial cells absolutely requires CaM [33] and it appears to also require Ca^2+ ^to sustain elevated level of activity [34].

Nitric oxide plays a critical role in the endothelial cell proliferation, migration, and tube formation, as well as increased vascular permeability, hypotension, and angiogenesis in vivo [35,36]. VEGF- and histamine-induced microvascular hyperpermeability are both mediated by a signalling cascade triggered by receptor binding and transduced by a serial activation of intracellular enzymes, including PLC, eNOS, soluble guanylate cyclase (sGC), and protein kinase G (PKG). Subsequently, the VEGF-activated NO-PKG pathway was linked to ERK1/2-mediated proliferation of cultured endothelial cells via phosphorylation and activation of the upstream p42/44 MAPK cascade component RAF by PKG [37].

### Thrombin, VEGF and Histamine mediated MLC activation

MLC of myosin II plays a critical role in controlling actomyosin contractility in both smooth muscle and nonmuscle cells [38-40]. MLC phosphorylation is regulated by the balance of two enzymatic activities, i.e., MLCK and myosin phosphatase (MYCP). MLCK is regulated by Ca^2+^/calmodulin and is believed to be a major kinase in both smooth muscle and nonmuscle cells. In addition, Rho-kinase (ROCK) can directly phosphorylate MLC in vitro [41]. MYCP is a holoenzyme composed of three subunits: a catalytic subunit of 38 kDa that was identified as protein phosphatase 1 (PP1) catalytic subunit δ-isoform (PP1Cδ) [42] and two noncatalytic subunits of 21 and 110-130 kDa [43,44]. The larger one, called myosin phosphatase targeting subunit 1 (MYPT1), binds to the catalytic subunit and targets it to MLC, providing substrate specificity [45]. ROCK and PKC have been proposed to mediate the inhibition of smooth muscle MYCP, leading to increased MLC phosphorylation in response to various agonists. Phosphorylation of the MYPT1 regulatory site (Thr695 in chicken MYPT1) by ROCK induces inhibition of MYCP activity [46]. Some experimental findings suggest that CPI-17, a soluble globular protein, is involved in PKC-dependent inhibition of MYCP and it has thus been considered as a specific inhibitor for MYCP [47].

Detail description of signaling cascades used in this model was provided the Additional File 3.

## Results and Discussion

### Model validation with experimental studies of the regulation of MLC activation, calcium release, and Rho activation by thrombin

**Figure 2:**
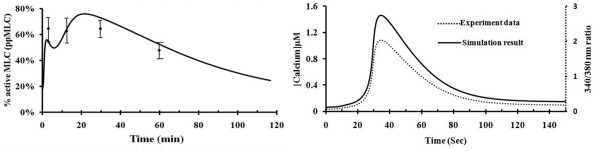
**Simulated time course and experimental data of thrombin-mediated MLC activation (left) and calcium release (right)**. Histamine and VEGF level set as zero values. refers to experimentally measured MLC activation at 2.5 min (68% ± 13%), 15 min (64% ± 13%), 30 min (67% ± 9%) and 60 min (48% ± 9%) (Ref 48).

Our simulation model was first validated by determining whether the simulation results were consistent with experimental observations of MLC activation and calcium release by the single mediator thrombin. Thrombin-mediated processes were investigated computationally by zeroing out the initial concentration of VEGF and histamine. It has been observed that MLC activation increases from low initial levels to 39% ± 2%, 66% ± 10, 68% ± 13%, 64% ± 13%, and 67 ± 9% of the MLC population at 30s, 60s, 2.5 min, 15 min, and 30 min after thrombin stimulation, respectively, which subsequently drops to 48% at 60 min [48]. The amplitude of MLC activation has been found to correlate linearly with the strength of endothelial cell contraction [49,50]. As illustrated in Figure 2 (Left), our simulated time-dependent MLC activation levels are in fair agreement with this observation (the simulation results for the first 20 min are also shown in Additional File 1, Figure S4). Our simulations showed that the amplitude of MLC activation reaches two peaks, the first at ~2.5 min and the main peak at ~30 min, which is compared to the observation that the levels of active MLC levels at 2.5 min and 30 min are higher than those at 15 min and 60 min [48]. Our analysis suggested that these two peaks arise primarily from the Ca^2+^-dependent and Rho GTPase/ROCK-dependent mechanisms, respectively, as described below.

Elevation in cytosolic Ca^2+ ^concentration ([51]) is a common initial response of endothelial cells to various changes such as the exposure to hormonal and inflammatory stimuli and variation of physical conditions [52]. Jeng *et al*. [53] have shown that the binding of thrombin and PAR-1 induces rapid calcium mobilization and increase of [Ca^2+^]i, with [Ca^2+^]i peaking at 30-40 s followed by a rapid drop. The simulated calcium release profile in Figure 2 (Right) exhibits a peak concentration at 38 s followed by a rapid decay, consistent with Jeng *et al*'s experiment results. The increased intracellular Ca^2+ ^influx is expected to enhance the binding of Ca^2+ ^to CaM, which subsequently activates MLCK to phosphorylate the MLC of myosin II. To evaluate which signalling event is primarily responsible for the large transient increase in the level of MLC activation (the first peak at ~2.5 min in the left Figure 2), we systematically varied the strength of protein-protein interactions upstream of MLC. As shown in Additional File 1, Figure S5, the first peak disappears when the Ca^2+^-dependent MLC activation (Reaction 73-86) was switched off, while that peak remains largely intact when the ROCK-dependent MLC activation and CPI-17-MYPT interactions were switched off (Reactions 57-58, 63-70, 99-102), Therefore, our analysis suggests that this Ca^2+^-dependent mechanism was primarily responsible for the large transient increase of the levels of MLC activation.

Thrombin induces a prolonged increase of endothelial permeability lasting for 1-1.5 hr. This prolonged elevated permeability is attributed to the activation of the small Rho GTPase and Rho kinase [54,55]. It has been found that Rho GTPase activation can be observed after 2 min and the elevated activation is maintained up to 60 min after thrombin stimulation, and the time course of Rho GTPase activation correlates well with the time course of MLC activation increase by experiment observation [54]. Our simulation result presented in Additional File 1, Figure S6 is consistent with this observation, which shows that the simulated Rho GTPase activation was maintained for 60 min. Rho GTPase activation induces MLC activation via both direct and indirect routes. Rho GTPase and ROCK directly activate MLC to subsequently induce the contraction of the non-muscle cell systems [41,56]. In the indirect route, ROCK inhibits myosin phosphatase activity by phosphorylating the myosin binding subunit (MBS) of myosin phosphatase [46], which increases the activation level of MLC, actomyosin interaction, stress fiber formation, and subsequent endothelial permeability. We studied whether these direct and indirect Rho GTPase -dependent mechanisms are primarily responsible for the sustained levels of MLC activation (the main peak at ~30 min in the left Figure 2) by systematically varying the protein-protein interactions upstream of MLC. As shown in Additional File 1, Figure S7, this peak remains largely intact when the Ca^2+ ^-dependent MLC activation and P-CPI-17-MYPT interaction (Reaction 57-58, 71-76) were switched off, but disappeared when the ROCK-dependent MLC activation (Reactions 63-70, 99-102) were switched off. Therefore, our analysis suggests that both the direct and indirect Rho GTPase -dependent mechanisms play an important role for the sustained levels of MLC activation.

### Model validation with experimental studies of MLC activation and ERK activation by VEGF

**Figure 3:**
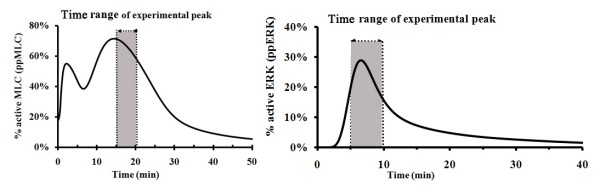
**Simulated time course and experimental result of VEGF-mediated MLC activation (left) and ERK activation (right)**. Thrombin and histamine level set at zero values. The shaded area in the left figure indicates the time range in which VEGF-induced vascular leakage reaches its peak in experimental studies (Ref 57). The shaded area in the right figure indicates the time range in which the amount of ERK-1/2 activation reaches maximum value after VEGF administration (Ref 58). The VEGF concentrations were set as 0.02 μM.

Our simulation model was also validated by determining whether the simulation results are consistent with experimentally observed regulation of MLC activation as well as ERK and MLCK activation by another mediator VEGF. These VEGF-mediated processes were simulated by using our model with thrombin and histamine switched off by setting their initial concentrations to zero values. It has been reported that injection of VEGF induces vascular leakage in 5 min, and the leakage peaks in 15-20 min and then diminishes after 30 min [57]. As shown in Figure 3 (Left), the simulated duration of MLC activation is about 30 min with the first peak at 2.5 min and the main peak at 15 min. The 15 min time range of the main peak of MLC activation is consistent with the reported 15-20 min time range for VEGF-induced vascular leakage to reach its peak [57]. While we have not found an experimental finding to support the true existence of the first peak exhibited by our simulation, it is noted that the time of the first peak matches the experimentally determined on-set time of VEGF-induced vascular leakage [57]. As described in the previous section, the first peak of MLC activation at ~2.5 min in Figure 3 (Left) was induced mainly by Ca^2+^-dependent mechanism. We further investigate which signalling event is primarily responsible for the main peak at ~15 min. As shown in Additional File 1, Figure S8. We found that this peak remained when NO-dependent MLC activation was switch off (Reactions 179-185) but disappeared when Ras-Raf-ERK-dependent MLC activation was switch off (Reactions 152-163). This suggests that the main peak is induced by Ras-dependent ERK activation. As shown in Figure 3 (Right), the simulated ERK activation peaks at about 7 min and decays within 25 min, which is consistent with the observation that the amount of phosphorylated ERK-1/2 reaches maximum value at 5-10 min after administration of VEGF and decreases back to the control level 30 min afterward [58].

### Model validation with experimental studies of MLC activation by histamine

The model was further validated by determining whether the simulation results are consistent with experimentally observed regulation of MLC activation by the third individual mediator histamine. This histamine-mediated process was simulated by using our model with thrombin and VEGF switched off by setting their initial concentrations to zero values. The simulation results in Additional File 1, Figure S9 indicated that histamine causes a transient increase of MLC activation that peaked at 2.5 min, which is consistent with the experimental finding that histamine induces a transient endothelial permeability peaked at 2-5 min [7]. Further investigation showed that this peak is primarily induced by Ca^2+^-dependent mechanism and the contribution from the NO-dependent ERK activation path is very small, as shown in Additional File 1, Figure S10 by switching off each individual path. Moreover, the contribution from the NO-dependent ERK activation path is much weaker compared with Ras-dependent ERK activation and MLC activation by the individual mediator VEGF.

### Comparison of the simulated thrombin-mediated IP3 and Ca^2+ ^release with that of an existing model

**Figure 4:**
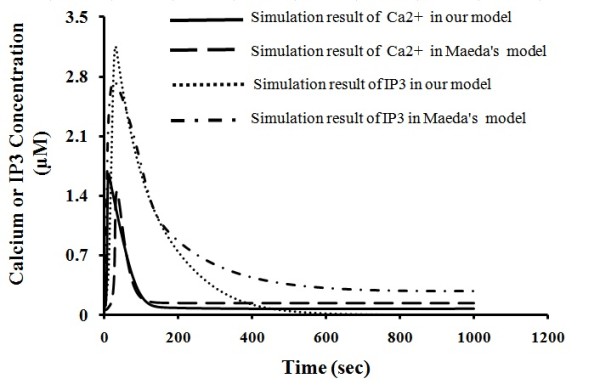
**Comparison of simulation result of Ca**^**2+ **^**and IP3 in our model and Maeda's model**.

The thrombin signalling cascade of our model is very similar to that of Maeda *et al*. that has been developed a computational model of thrombin-regulated ROCK pathway [18]. Hence, it is appropriate to compare the simulation results of our model with Maeda's model. In their studies, they measured and simulated thrombin-mediated IP3 and Ca^2+ ^release. We therefore compared our simulated IP3 and Ca^2+ ^release with their results. As shown in Figure 4, our simulation showed essentially the same transit IP3 release and Ca^2+ ^release patterns as those presented in Maeda's studies.

### Simulation of the effects of thrombin receptor PAR-1 over-expression on thrombin-mediated MLC activation

**Figure 5:**
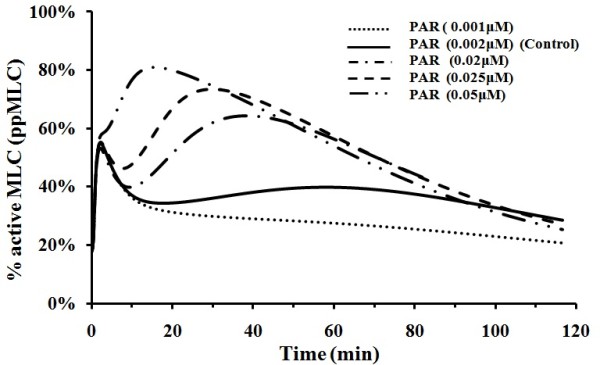
**MLC activation at different PAR-1 concentrations**. Thrombin level set at 0.05 μM with histamine and VEGF level at zero value. The physiological concentration range of PAR-1 is 0.002 - 0.02 μM.

PAR-1 is the major thrombin-activated receptor involved in platelet aggregation, endothelial permeability, and tumor cell migration. Activated PAR-1 is coupled via several members of the heterotrimeric G-proteins, Gα12/13 and Gαq, to transduce a substantial network of signalling pathways [26]. It has been reported that during atherogenesis, PAR-1 expression is enhanced in regions of inflammation associated with macrophage influx, smooth muscle cell proliferation, and an increase in mesenchymal-like intimal cells [59]. It is of interest to quantitatively evaluate the effects of PAR-1 elevation on thrombin-mediated MLC activation. We further used our model to simulate thrombin mediated MLC activation at different PAR-1 levels with VEGF and histamine switched off [60]. Our simulation results, in Figure 5, showed that PAR-1 at elevated levels significantly increases the amplitude of MLC activation and reduces the time for MLC activation to reach the main peak. There is a direct correlation between the level of PAR-1 expression and the degree of invasiveness of breast carcinoma cell lines [61], in which endothelial permeability is one of the prerequisites for cancer invasiveness as it facilitates cell transmigration and plasma accumulation in the matrix to support new vessel formation [60]. Therefore, this experiment indicated that PAR-1 over-expression leads to enhanced endothelial hyper-permeability, and our simulation results are in good agreement with this experimental finding.

### Simulation of the effects of Rho GTPase and ROCK over-expression on thrombin-mediated MLC activation

**Figure 6:**
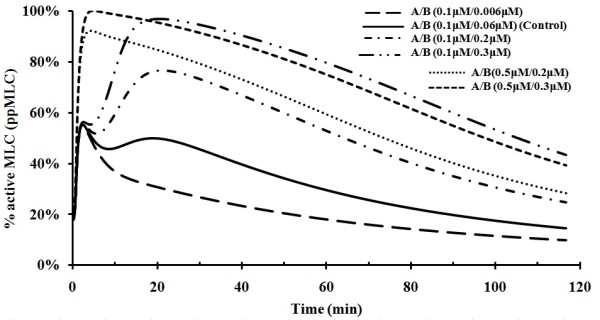
**MLC activation at different Rho GTPase (A) and ROCK (B) concentrations**. Thrombin level set at 0.05 μM with histamine and VEGF level at zero value. The physiological concentration range of ROCK is 0.06 - 0.3 μM.

Rho GTPase and ROCK in endothelial cells have been found to be elevated in hypoxia [62]. Over-expression of dominant activated Rho GTPase/ROCK in NIH3T3 cells results in an increase of MLC activation [46]. Over-expressed ROCK in human brain microvascular endothelial cells has been found to induce endothelial permeability and to significantly increase the transmigration rate of NCI-H209 cells through the human brain microvascular endothelial cells [63]. The effects of elevated Rho GTPase and ROCK on thrombin-mediated MLC activation were quantitatively evaluated by using our model with VEGF and histamine switched off. As shown in Figure 6, an increased ROCK level with Rho GTPase at control level significantly enhanced the amplitude of activation of MLC in a dose-dependent manner. When ROCK and Rho GTPase levels were simultaneously elevated, the amplitude of MLC activation was significantly increased and the time to reach the activation peak was reduced. Rho GTPase and ROCK are abundant in lymph nodes with metastasis, and the ability to enter either blood or lymphatic vasculature is necessary for tumor cells to metastasize to distant sites [64]. Furthermore, Rho GTPase and ROCK reportedly are required in both endothelial and migrating cells for them to cross the vascular endothelium [65,66]. Thus, by quantifying the effect of Rho GTPase/ROCK, we can gain more insight into the mechanism of sustained MLC activation, which may aid the search for and evaluation of new therapeutic strategies for the prevention and treatment of endothelial hyper-permeability and cancer metastasis-related diseases.

### Simulation of effects of VEGF and VEGFR2 over-expression on VEGF-mediated MLC activation

VEGFR2 is recognized as the principal mediator of physiological and pathological effects of VEGF on endothelial cells, which include proliferation, migration, survival, and permeability [67]. The expression of VEGF and VEGFR2 in endothelial cells has been found to be elevated in oxidative stress [68], type 1 leprosy reaction [69], and during diabetes to induce microvascular complications, especially diabetic retinopathy [70]. Over-expression of VEGF and VEGFR2 has been shown to correlate with increased risk of metastatic disease and overall poor prognosis in different carcinomas [71]. Apart from their primary functions in angiogenesis, the roles of VEGF and VEGFR2 in metastasis likely involve the regulation of endothelial permeability to facilitate cell transmigration and plasma accumulation in the matrix in support of new vessel formation [72]. The effects of VEGF and VEGFR2 over-expression on VEGF-mediated MLC activation were quantitatively evaluated by using our model with thrombin and histamine switched off.

**Figure 7:**
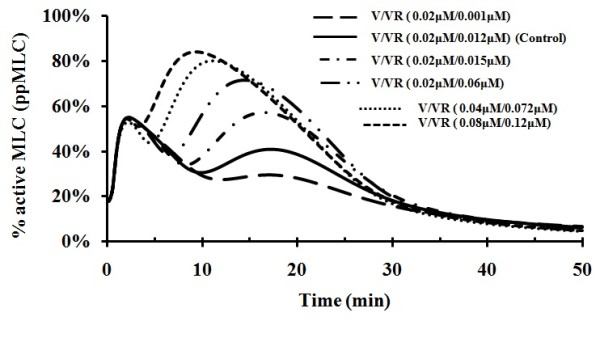
**MLC activation at different VEGF(V) and VEGFR2 (VR) concentrations**. Thrombin and histamine levels set at zero values. The physiological concentration range of VEGFR is 0.012-0.072 μM.

As shown in Figure 7, the increased amount of VEGFR2 with VEGF at control level significantly enhanced MLC activation. For instance, the small increase of VEGFR2 concentration from 0.010 to 0.012 μM increased the amplitude of the main peak of MLC activation by 15%, suggesting that MLC activation was very sensitive to VEGFR2 concentration. When VEGF and VGEFR2 levels were simultaneously increased, the amplitude of MLC activation was further increased by a significant amount with respect to that when only VEGFR2 was over-expressed. This is consistent with the observed correlation of VEGF and VEGFR2 over-expression with increased risk of metastatic disease and overall poor prognosis in different carcinomas [71].

### Simulation of synergistic activation of MLC by thrombin and histamine

**Figure 8:**
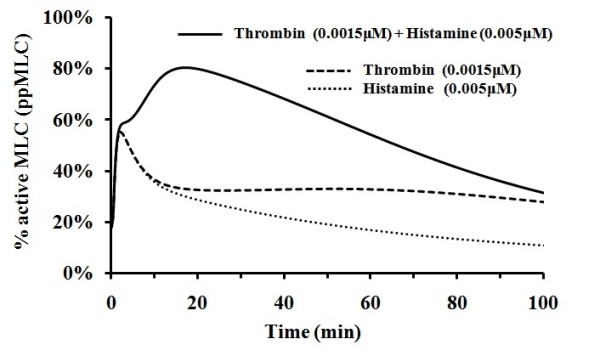
**MLC activation induced by combination of thrombin and histamine stimuli**. The solid, long dashed, and dot line corresponds to the activation by thrombin and histamine with VEGF level set at zero value, by thrombin with histamine and VEGF level set at zero values, and by histamine with thrombin and VEGF level set at zero value respectively.

It has been reported that combination of low concentrations of stimuli of thrombin and histamine induces more significantly enhanced endothelial permeability than the simple sum of the permeability change induced by each mediator alone [73]. The effect of the combination of low concentrations of thrombin and histamine on MLC activation was explored by using our model with the third mediator VEGF switched off. As illustrated in Figure 8, from 10 min to 50 min after stimulation with combination of 0.0015 μM thrombin and 0.0050 μM histamine, the amplitude of MLC activation reached levels of >65%, which is greater than the simple sum of <35% and <22% when only one individual mediator, thrombin and histamine, respectively, was switched on. Therefore, our simulation results indicated a synergistic effect of histamine and thrombin, in agreement with observations [73]. Moreover, the levels of MLC activation induced by these low concentrations of thrombin and histamine are comparable to (higher than) those induced by individual mediator thrombin and histamine at concentrations of 0.0500 μM and 0.005 μM, respectively, which suggests that the synergistic effect is at a substantial level.

The level of synergistic effect can be more clearly revealed by the comparison of the areas under the thrombin and histamine induced MLC activation curve with those of thrombin-induced and histamine-induced MLC activation curves at different 10 min time intervals in Figure 8, which are provided in Table 1. In particular, the level of synergistic effect can be reflected by the difference between the area under the thrombin and histamine induced curve and the simple sum of the areas under the thrombin-induced and histamine-induced curves, with positive values corresponding to synergistic effect (better than simple sum of thrombin-induced and histamine-induced activation). From Table 1, the largest synergistic effect occurs in the 10-20 min, 20-30 min and 30-40 min time ranges with net area gain of 1.3, 1.8 and 1.5 (corresponding to an average of 13%, 18% and 15% more amount of activated MLC with respect to that of simple sum of thrombin-induced and histamine-induced activation).

**Table 1:** Comparison of the areas with respect to different time ranges in Figure 8

MLC activation curve	Area under MLC activation curve with respect to different time range (time unit:min)							
	
	0-10	10-20	20-30	30-40	40-50	50-60	60-70	70-80
Curve 1:Histamine + Thrombin induced activation	5.9	7.8	7.6	6.9	6.1	5.2	4.7	4.0

Curve 2:Histamine-mediated activation	4.3	3.1	2.6	2.3	2.0	1.6	1.4	1.3

Curve 3:Thrombin-mediated activation	4.4	3.4	3.2	3.1	3.1	3.0	3.0	2.9

Simple sum of curve 2 and 3	8.7	6.5	5.8	5.4	5.0	4.7	4.5	4.3

Area difference between curve 1 and simple sum of curve 2 and 3	-2.8	1.3	1.8	1.5	1.1	0.6	0.2	-0.3

Comparison of the areas under the thrombin and histamine induced MLC activation curve with those of thrombin-induced and histamine-induced MLC activation curves with respect to different time ranges in Figure 8.

As shown in Figure 8, the synergistic effect at low concentrations of thrombin and histamine only occur during the time range from 10 min to 50 min. Before and after this time range, the level of MLC activation by thrombin + histamine is less than the simple sum of that by thrombin and histamine alone. The less than additive effect during the first 10 min is primarily due to the time-dependent behavior of MLC activation by the Ca^2+^-dependent signalling cascade. The transient MLC activation curve by the Ca^2+^-dependent cascade is largely the same for the thrombin, histamine, and thrombin + histamine mediated processes (Additional File 1, Figure S11, S12 and S13, solid line). It is thus not difficult to understand that the simple sum of the level of MLC activation by thrombin and histamine alone is superficially larger than that by thrombin + histamine. The less than additive effect after 50 min is primarily due to the variation of time-dependent behavior of MLC activation by the ROCK-dependent signalling cascade. The level of MLC activation slowly rises to significant levels without decay in the presence of thrombin alone for up to 100 min (Additional File 1, Figure S11, dotted and dash-dotted line). On the other hand, the MLC activation level rises slowly to moderate levels without decay in the presence of histamine alone (Additional File 1, Figure S12). In contrast, the MLC activation level quickly rises to high levels and rapidly decays to low levels after 50 min in the presence of thrombin + histamine, the signalling strength thus becomes less than additive after 50 min (Additional File 1, Figure S13, dash-dotted line ).

The underling mechanism of the significant synergistic effect during 10-50 min time period can be elucidated from the perspective of network regulation. MLC activation is regulated by at least four signalling cascades Ca^2+^-dependent, CPI-17-dependent, NO-dependent, and ROCK-dependent cascades (Figure 1). As shown in Additional File 1, Figure S11, S12, and S13, The MLC activation curve induced by the Ca^2+^-dependent cascade is roughly the same for the thrombin, histamine and thrombin + histamine mediated processes. The level of MLC activation induced by the CPI-17-dependent, NO-dependent cascade in the presence of thrombin + histamine is close to the simple sum of that in the presence of thrombin and histamine alone. While the MLC activation induced by the ROCK-dependent cascade in the presence of thrombin + histamine is at significantly higher levels and shows more transient pattern than that in the presence of thrombin and histamine alone. These differences in signalling behavior lead to synergistic effect within 10 min to 50 min time range.

The different signalling behavior of the Rho-ROCK signalling stimulated by different mediators or mediator combinations primarily arises from the dynamics of ROCK activation [74]. The kinase activity of ROCK is off when ROCK is intra-molecularly folded. ROCK can be activated only after it is unfolded by the binding of Rho GTPase to its Rho-binding domain to disrupt the auto-inhibitory interaction, which subsequently allows such proteins as Rho GTPase and PKG to activate ROCK at phosphorylation site. Hence, in the presence of thrombin + histamine, thrombin-activated Rho GTPase unfolds ROCK to allow histamine-activated PKG to activate ROCK thereby enhancing the level of ROCK activation in combination with thrombin-mediated Rho GTPase activation of ROCK. When stimulated by histamine or VEGF alone, ROCK is in the inactive state and does not contribute to MLC activation. When stimulated by thrombin alone, ROCK is activated only by Rho GTPase without the contribution from PKG, leading to a slower increase and lower peak strength of MLC activation than that in the presence of thrombin + histamine. Such an integrated communication network is expected to enable fine tuning of the strength and duration of MLC activation, thereby enabling fine regulation of physiological responses, including synergistic or more complex effects. Network models have suggested that partial inhibition of a surprisingly small number of targets can be more efficient than the complete inhibition of a single target [75]. Experimental and simulation studies of synergistic effects of thrombin and histamine on endothelial monolayer permeability may provide useful information for developing multi-target drugs against endothelial permeability and related diseases [75].

### Prediction of the collective regulation of MLC activation by thrombin and VEGF

Our simulation model was further used to study the collective regulation of MLC activation by thrombin and VEGF, with a particular focus on whether or not thrombin and VEGF synergistically activate MLC in certain time ranges. Systemic activation of blood coagulation is often present in cancer patients, and thrombin generated during thrombosis can augment malignant phenotypes by activating tumor cell adhesion to platelets and endothelial cells, enhancing tumor cell growth and metastasis, and stimulating tumor cell angiogenesis [1]. Moreover, thrombin promotes VEGF secretion and platelet activation, thus causing a mutual stimulation between endothelial cells and cancer cells [76,77]. Therefore, the collective effect of thrombin and VEGF on MLC activation and subsequently endothelial hyperpermability may have substantial influence on the tumor growth and metastasis process in cancer patients with blood coagulation near and at the tumor sites [78].

**Figure 9:**
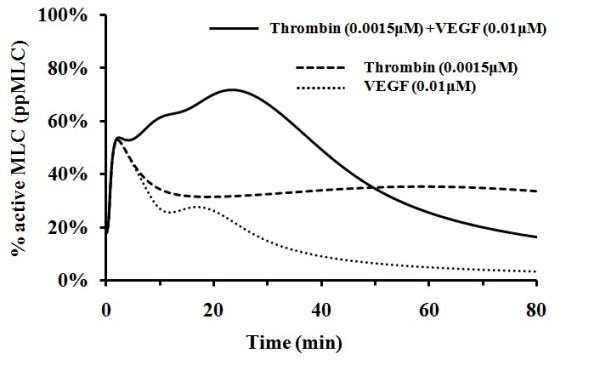
**MLC activation induced by the combination of thrombin and VEGF stimuli**. The solid, long dashed, and dot line corresponds to the activation by thrombin and VEGF with histamine level set at zero value, by thrombin with histamine and VEGF level set at zero value, and by VEGF with thrombin and histamine level set at zero values.

As shown in Figure 9, from 15 min to 30 min after stimulation with combination of 0.002 μM thrombin and 0.010 μM VEGF, the amplitude of MLC activation reached levels of >62%, which is greater than the simple sum of <32% and <28% when only one individual mediator, thrombin and VEGF, respectively, was switched on. Therefore, our simulation results indicated a synergistic effect of histamine and VEGF on MLC activation. The level of synergistic effect can be reflected by the difference between the area under the thrombin and VEGF induced curve and the simple sum of the areas under the thrombin-induced and VEGF-induced curves in Figure 9, which are shown in Table 2. From Table 2, the largest synergistic effect occurs in the 20-30 min time range with net area gain of 1.8 corresponding to an average of 18% more amount of activated MLC with respect to simple sum of thrombin-induced and VEGF-induced activation. The high level MLC activation by thrombin and VEGF likely has significant impact on the promotion of cancer metastasis in the cancer patients with blood coagulation near and at the tumor sites. These patients may be more effectively treated by combinations of drugs targeting the VEGF and thrombin signalling pathways [78].

**Table 2:** Comparison of the areas with respect to different time ranges in Figure 9

MLC activation curve	Area under MLC activation curve with respect to different time range (time unit:min)							
	
	0-10	10-20	20-30	30-40	40-50	50-60	60-70	70-80
Curve 1:Thrombin + VEGF induced activation	5.0	6.2	6.7	5.1	3.4	2.4	1.9	1.4

Curve 2:VEGF-mediated activation	4.1	3.5	2.9	3.2	3.3	3.4	3.4	3.1

Curve 3:Thrombin-mediated activation	4.0	2.6	2.0	1.1	0.05	0.5	0.4	0.3

Simple sum of curve 2 and 3	8.1	6.1	4.9	4.3	3.35	3.9	3.8	3.4

Area difference between curve 1 and simple sum of curve 2 and 3	-3.1	0.1	1.8	0.7	0.05	-1.5	-1.9	-2.0

Comparison of the areas under the thrombin and VEGF induced MLC activation curve with those of thrombin-induced and VEGF-induced MLC activation curves with respect to different time ranges in Figure 9.

### Prediction of the effect of CPI-17 over-expression on MLC activation in the presence of lower concentration of thrombin, histamine and VEGF

**Figure 10:**
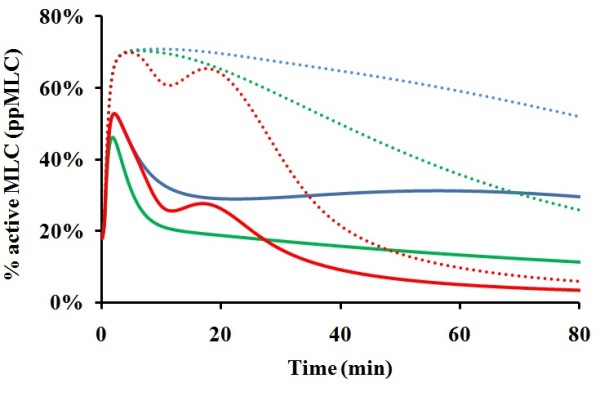
**Prediction of the effect of CPI-17 over-expression on MLC activation at low concentration of stimuli**. Solid and doted lines correspond to the activation by default (CPI-17 = 0.08 μM) and elevated (CPI-17 = 0.4 μM) concentration of CPI-17. Blue line refers to thrombin stimuli, green line refers to histamine stimuli, and red line refers to VEGF stimuli respectively.

CPI-17 inhibits MYCP to hinder its dephosphorylation of MLC, leading to increased MLC activation [79]. Altered CPI-17 level is associated with smooth muscle-related diseases, such as intestinal bowel disease [80], asthma [81], pulmonary hypertension [82] and diabetic dysfunction of smooth muscle [83]. It is of interest to evaluate the effect of CPI-17 over-expression on MLC activation, particularly at lower level of thrombin, histamine and VEGF. In this work, CPI-17 over-expression was simulated by 5-fold increase of its level from 0.08 μM to 0.4 μM [84]. Each of the thrombin-, histamine- and VEGF- mediated processes was simulated by setting the concentration of thrombin, histamine and VEGF set at lower value of 0.0015 μM, 0.005 μM and 0.01 μM respectively with the other two mediators switched off by setting their initial concentrations to zero values. As shown in Figure 10, CPI-17 over-expression significantly strengthened and prolonged MLC activation to the levels higher than those at normal CPI-17 level and normal concentration of thrombin, histamine and VEGF respectively [85].

## Conclusion

Thrombin, VEGF, and histamine are hallmarks of endothelial hyper-permeability, which perform their regulatory roles individually and collectively under different disease conditions, and with different dynamic profiles. Thrombin and VEGF can increase microvascular permeability ~50,000 times more potently than histamine [86]. Thrombin, VEGF, and histamine induce prolonged (1-1.5 hr), intermediate (15-20 min) and transient (~5 min) increases of endothelial permeability, respectively. An integrated simulation model that includes the signalling of all these hallmark mediators enables more comprehensive analysis of the signalling processes involved in different disease processes and regulated by different combinations of these mediators.

Based on published models of relevant signalling, we developed an integrated mathematical model including the signalling pathways of all three of these mediators. Simulation results from our model were consistent with available experimental data of signalling mediated by both individual mediators and combinations of two mediators, and could be used to interpret the sustained and transient phases of MLC activation. Our model was able to predict the effects of altered pathway components and synergistic combination of multiple mediators, some of which are consistent with experimental findings [73]. Similar to the published models of other pathways, our model can potentially be used to identify important disease genes through sensitivity analysis of signalling components [87]. Our model may also be extended to emphasize other components to facilitate further investigation of the effects of different mediators, cascades, and cross-talk on endothelial permeability and related diseases.

## Methods

### Model Development

One of the most commonly used approaches to model biological systems is that of ODEs. In general, a differential equation can be used to describe the chemical reaction rate that depends on the change of participating species over time. The temporal dynamic behavior of molecular species in the biological signalling pathway network can be captured by a set of coupled ODEs. Our pathway model is illustrated in Figure 1. Thrombin, VEGF and histamine induced MLC activation, as well as Ca^2+^-dependent and ROCK-dependent activation of MLC, were included in the model. The constituent molecular interactions, their kinetic constants and molecular concentrations are described in detail in Additional File 2. The ODEs for these interactions were derived based on mass-action laws with interaction rate constants defined by the forward and reverse rate constants Kf and Kb or turnover number Kcat for enzymatic reactions derived from published models [88,89] and other literature. Our simulation model contains 200 equations and interactions and 185 distinct molecular species, characterized by 319 kinetic parameters and 48 initial molecular concentrations. These ODEs were then solved by using the Dormand-Prince pair based Ode45 solver of Matlab with the absolute tolerance of 1.0E-6 and relative tolerance 0.0010. A Systems Biology Markup Language (SBML) version of our model is provided at http://bidd.nus.edu.sg/group/Supplement.htm, and uploaded into the BioModels [90] and KDBI [91] databases (Additional File 4).

### Collection and estimation of kinetic parameters

The types of parameters used in our model are parameters governing protein-protein interactions and catalytic activities. The published simulation studies have found that most parameters are robust and moderate changes do not significantly alter the overall pathway behavior [15,16,18]. Apart from the use of the parameters of the published simulation models, additional parameters were obtained from the literature based on the widely used assumption that parameters measured in vitro and in some cell lines are generally applicable in most cases. For those protein-protein interactions without available parameters, their parameters were putatively estimated from the known parameters of the relevant interacting domain profile pairs [92,93] or other interacting protein pairs of similar sequences. As a biological network is believed to be robust, and protein-protein binding interactions for proteins in similar families that mediate similar types of biochemical reactions (such as Ras and Rho) often differ within a 10-fold range, the values of kinetic parameters obtained from previous models were optimized within this range.

The parameters of the protein-pairs not available from previous models were obtained by the following procedure: The first step in finding the parameters of a specific protein-pair is to search protein-pairs that are both with available parameters and with each individual protein similar in sequence with the respective protein of the studied protein-pair. If one or more such protein-pairs are found, then the average values of the parameters of these protein-pairs are used as the initial parameters of the studied protein-pair, which are further optimized in ± 10-fold range with respect to experimental data. For instance, the parameters for the Rho activation cycle were obtained from the Ras activation cycle and were further optimized within a 10-fold range. The cycle of optimization and validation was repeated in order to obtain simulated results that agreed well with known experimental trends. If no such protein-pair is found, we proceed to the second step to search protein-pairs that are both with available parameters and with each individual protein belonging to the same domain family of the respective protein of the studied protein-pair. If one or more such protein-pairs are found, then the average values of the parameters of these protein-pairs are used as the initial parameters of the studied protein-pair, which are further optimized in ± 10-fold range with respect to experimental data.

The parameters of RhoGAP and PKC related protein-pairs were determined by the first and second step. The parameters of 14 protein-pairs could not be determined by the first and second step due to lack of experimental data and relevant protein-pairs with known parameters. These parameters were determined by using the trust regions algorithm [94] to fit the simulation results to the experimental data of RAS, ERK, MYPT and CPI-17 activation curves [95-97]. Additional File 1, Figure S14 shows the fitting curve against experimental RAS activation data. The level of fitting is based on the least-squares method and the fitting process proceeds in iterations until the R-square value is >0.6[98]. In each iteration, the parameter values derived from the previous iteration were used as the starting parameters for further optimization.

### Model Optimization, Validation and Parameter Sensitivity Analysis

Mathematical models developed by 'one-set-fits-all' generic parameters need not reproduce quantitative behavior in all systems, but may be able to reproduce the behavior or trend for specific systems. For instance, a mathematical model developed for a biological pathway from parameters obtained experimentally from one cell type can behave slightly different in another cell types. Differences in model behavior between cell types can be due to the presence or absence of crosstalk (i.e., differences in model topology) and variation in effective values of kinetic parameters. Hence, in this study, we developed a generic model of the thrombin-, VEGF-, and histamine-MLC signalling pathway to investigate the role of these three main mediators in regulating MLC activation. The simulated results are represented as trajectories of concentrations of chemical species with respect to time that are validated against available experimental data. If the trend or dynamic behavior of a particular reactant or product behaves in such a way that is consistent with the experimental data, then the model is said to be reasonable and can be used to analyze and predict unknown biological phenomena within some difficulty to define range of conditions. If the simulation results are not in agreement with known experimental facts, then the model has to be revisited to examine possible errors, such as incorrect interaction kinetics or values of kinetic parameters. Optimized parameters obtained from previous mathematical models are not necessarily optimized in a new study as the scope of these models can be different. The cycle of optimization and validation are repeated in order to obtain simulated results that agree well with known experimental trends.

The sensitivity of the simulation results with respect to the optimized and other parameters need to be systematically analyzed to determine if the model is sufficiently robust to be able to analyze and predict the true dynamic behavior of biological networks without the artifact of parameters. Differential analysis of parameter sensitivity, also referred to as the direct method, was utilized to compute the time-dependent sensitivities of all the species states with respect to each parameter values in the model [99]. Complex-step derivative approximation [100] was used to calculate numerical derivatives of the reactions in the model to achieve near analytical accuracy, robustness and easy implementation. We used sensitivity analysis function of Matlab to conduct sensitivity analysis. The sensitivity value of ppMLC with respect to all parameters in the model was provided in Additional File 5 and Additional File 1, Figure S15. As shown in Additional File 1, Figure S15, only 14 (4%) kinetic parameters including CPI-17, PKC and ROCK related reactions showed some sensitivity in affecting the output. The majority of the parameters are insensitive in affecting the output. Thus, our model can be considered as sufficiently robust.

## Competing interests

The authors declare that they have no competing interests.

## Authors' contributions

YZC and BT planned the project and revised the manuscript; XNW performed the analysis; BCH, JXZ, XHL, BCL, CYT, YYJ helped with the analytical work and the writing process. All authors read and approved the final version of the manuscript.

## Supplementary Material

Additional file 1 **Supplementary Figures**. Detailed pathway map (Figure S1-S3); Simulated time course and experimental data(Figure S4, S6, S9);Simulated time course of MLC activation in terms of different components (Figure S5, S7, S8, S10-S13); Fit to experimental data for Ras activation (Figure S14); Parameter sensitivity analysis (Figure S15). Click here for file

Additional file 2 **Supplementary Table**. Chemical reactions and related kinetic parameters used in this model. Click here for file

Additional file 3 **Supplementary Model Description**. Detail description of signaling cascades used in this model. Click here for file

Additional file 4 **SBML file for the model**. A Systems Biology Markup Language (SBML) version of our model was provided in Additional File 5. Click here for file

Additional file 5 **Sensitivity analysis data**. The sensitivity value of ppMLC with respect to all parameters in the model was provided in Additional File 4. Click here for file
