# Supplementary material for: An Integrated Mathematical Model of Thrombin-, Histamine-and VEGF-Mediated Signalling in Endothelial Permeability
Source: BMC Syst Biol. 2011 Jul 15;5:112. doi: 10.1186/1752-0509-5-112 (PMC3149001; doi:10.1186/1752-0509-5-112)
Supplement: Additional file 1 — Supplementary Figures. Detailed pathway map (Figure S1-S3); Simulated time course and experimental data(Figure S4, S6, S9);Simulated time course of MLC activation in terms of different components (Figure S5, S7, S8, S10-S13); Fit to experimental data for Ras activation (Figure S14); Parameter sensitivity analysis (Figure S15). [file 1752-0509-5-112-S1.DOC]

**Supplementary Figures**

**Figure S1 The detailed pathway map of the thrombin-mediated signalling component of our integrated pathway simulation model. ROCK (f) and ROCK (o) refer to ROCK in folded and open conformation respectively.**

**PLCβ**

**Rho GEF**

**Gα12,13**

**PAR-1**

**Gq**

**Rho GTP**

**ROCK (f)**

**RhoGDI**

**RhoGAP**

**DAG**

**PKC**

**MLCK**

**CPI-17**

**PP-MLC**

**MLC**

**P-MYPT**

**MYPT**

**P-CPI-17**

**RGS**

**ROCK (o)**

**pROCK**

**Thrombin**

**Figure S2 The detailed pathway map of the histamine-mediated signalling component of our integrated pathway simulation model.**

**H1R**

**PLCβ**

**DAG**

**PKC**

**CPI-17**

**P-CPI-17**

**PP-MLC**

**MLC**

**P-MYPT**

**MYPT**

**MLCK**

**Gq**

**eNOS**

**PKG**

**CaM**

**Raf**

**MEKK**

**ERK**

**NO**

**Histamine**

**Figure S3 The detailed pathway map of the VEGF-mediated signalling component of our integrated pathway simulation model.**

**VEGFR-2**

**SOS**

**RasGTP**

**Raf**

**MEKK**

**GC**

**PLCγ**

**DAG**

**PKC**

**CPI-17**

**P-CPI-17**

**eNOS**

**NO**

**ERK**

**PKG**

**PP-MLC**

**MLC**

**P-MYPT**

**MYPT**

**MLCK**

**CaM**

**VEGF**

**Figure S4 Simulated time course and experimental data of thrombin-mediated MLC activation in the first 20 min.
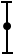
 denotes experimentally measured MLC activation at 30s (39%±2), 60s (66%±10%), 2.5min (68%±13%).**

**
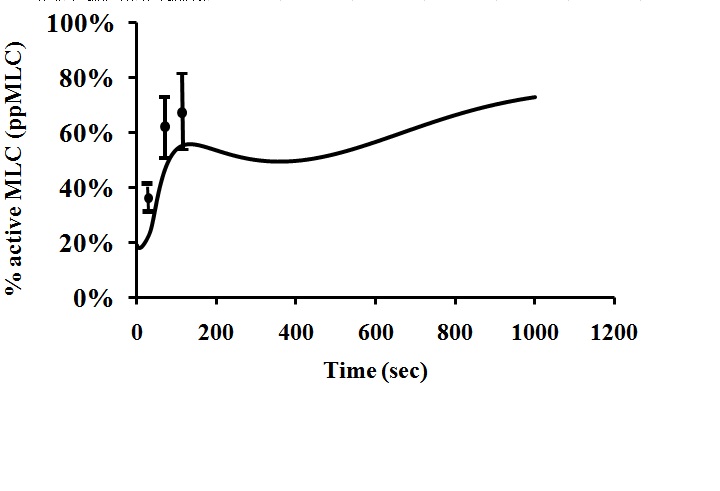
**

**Figure S5 Simulated time course of thrombin-mediated MLC activation in terms of different components. The curve , and represents the signaling from the complete pathway (Control), the Ca2+-dependent component (with ROCK-dependent MLC activation and P-CPI-17-MYPT interaction switched off, Reactions 59-60, 65-72, 101-104), and the non- Ca2+-dependent component (with Ca2+ -dependent MLC activation switched off, Reactions 73-88) respectively.**


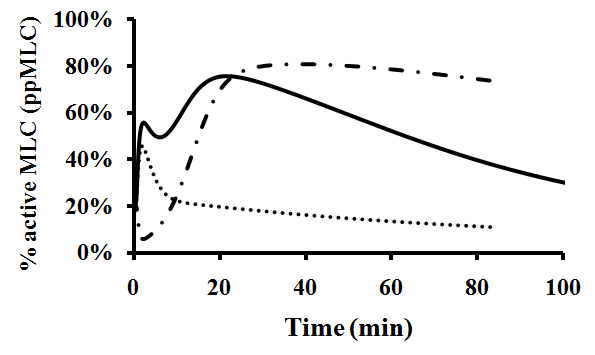


**Figure S6 Simulated time course and experimental results of thrombin-mediated Rho GTPase activation in units of percentage of initial Rho concentration.**


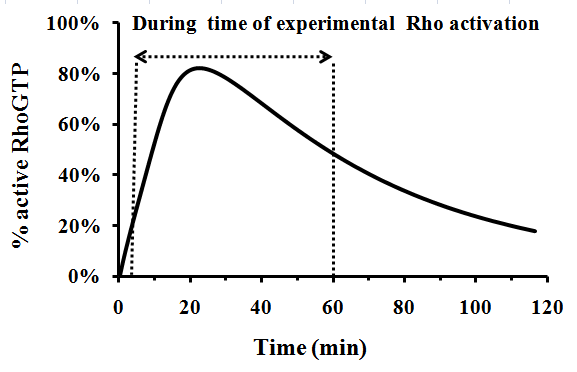


**Figure S7 Simulated time course of thrombin-mediated MLC activation in terms of different components. The curve , and represents the signaling from the complete pathway (Control), ROCK-dependent component (with Ca2+ -dependent MLC activation and P-CPI-17-MYPT interaction switched off, Reactions 59-60, 73-78), and the non- ROCK-dependent component (with ROCK-dependent MLC activation switched off, Reactions 65-72, 101-104) respectively.**


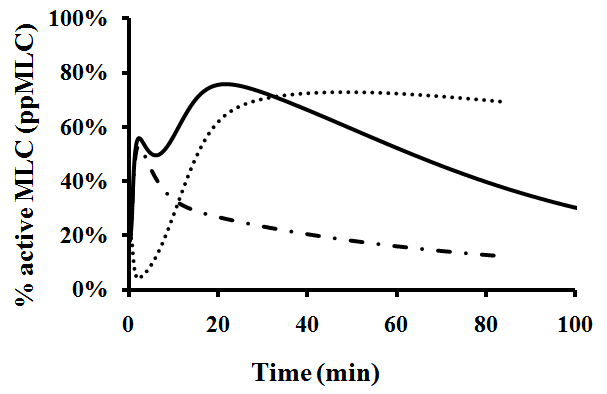


**Figure S8 Simulated time course of VEGF-mediated MLC activation in terms of different components. The curve, and represents the signaling from the complete pathway (Control), non-ERK-dependent component (with Ras-Raf-ERK-dependent MLC activation switched off, Reactions 154-165), and the non- NO-dependent component (with NO- dependent MLC activation switched off Reactions 179-185) respectively.**

**
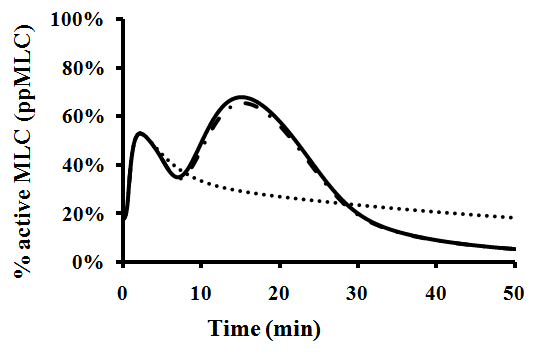
**

**Figure S9 Simulated time course and experimental result of Histamine-mediated MLC activation in units of percentage of initial MLC concentration with thrombin and VEGF level set at zero values. The shaded area indicates the time range in which histamine has been experimentally found to induce a transient endothelial permeability. The histamine concentrations were taken as 0.005µM.**

**
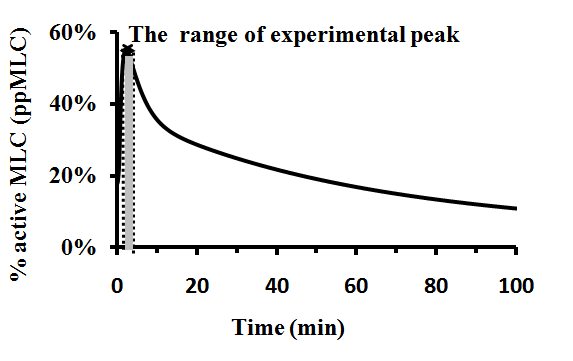
**

**Figure S10 Simulated time course of Histamine-mediated MLC activation in terms of different components. The curve , and represents the signaling from the complete pathway (Control), non- Ca2+-dependent component (with Ca2+-dependent MLC activation switched off, Reaction 73-88), and the non-NO-dependent component (with NO- dependent MLC activation switched off, Reaction 179-185 ) respectively.**

**
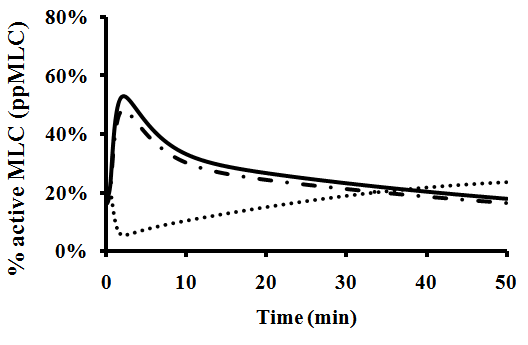
**

**Figure S11 The contribution of Ca2+- dependent, ROCK-dependent and CPI-17-dependent signaling cascade to thrombin-mediated MLC activation at low concentration of thrombin (0.0015 µM).**

**
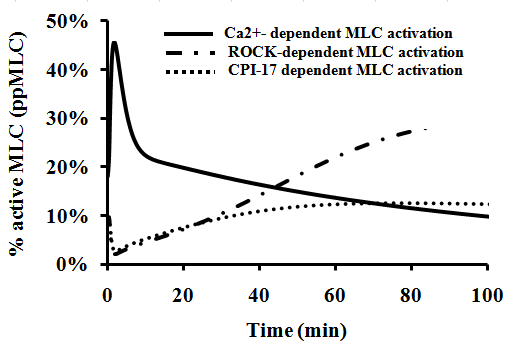
**

**Figure S12 The contribution of Ca2+- dependent, NO-dependent and CPI-17-dependent signaling cascade to histamine-mediated MLC activation at low concentration of histamine (0.005 µM).**

**
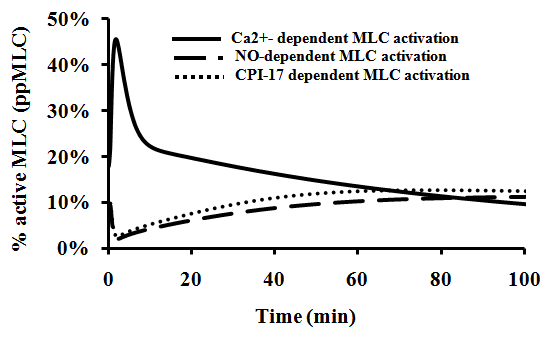
**

**Figure S13 The contribution of Ca2+- dependent, NO-dependent and CPI-17-dependent cascade to thrombin + histamine mediated MLC activation at low concentration of thrombin (0.0015 µM) and histamine (0.005 µM).**

**
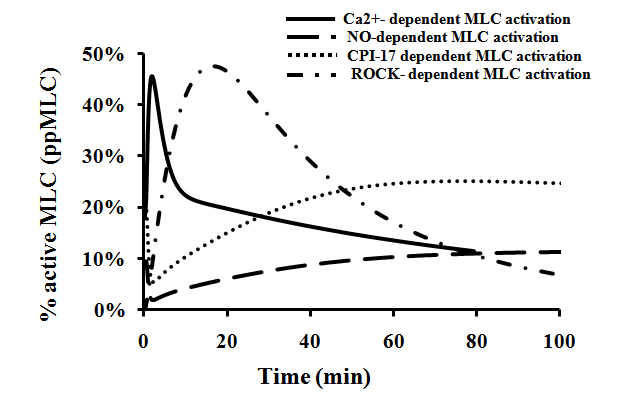
**

**Figure S14 Fit to experimental data for Ras activation.**

**
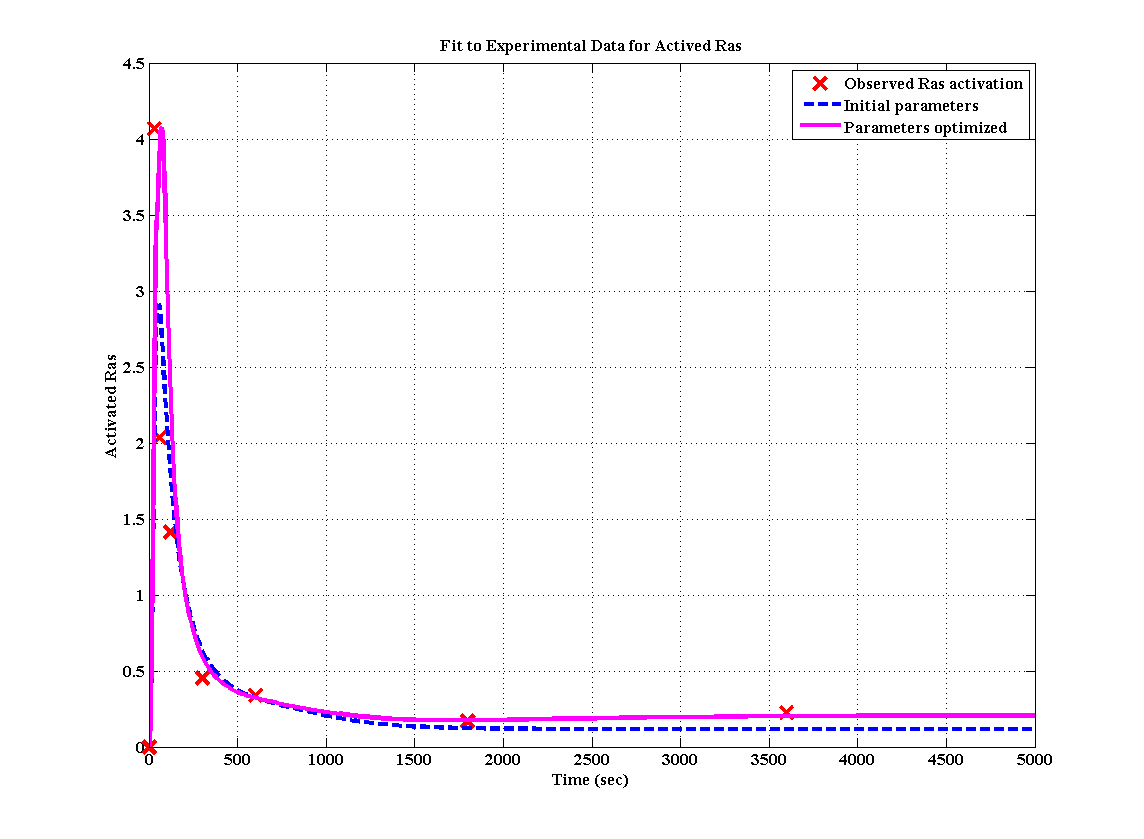
**

**Figure S15 Parameter sensitivity analysis (The detail data are provided in the supplementary material 2)**

**
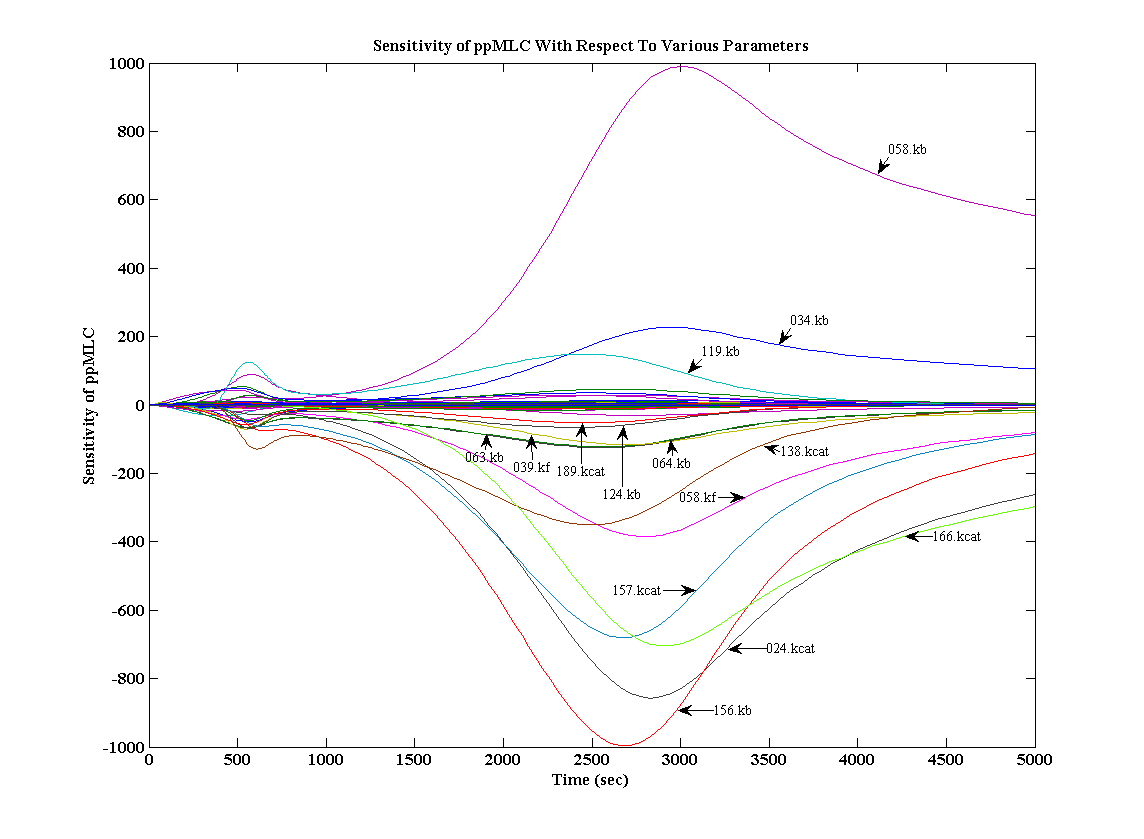
**
