# Supplementary material for: An Integrated Mathematical Model of Thrombin-, Histamine-and VEGF-Mediated Signalling in Endothelial Permeability
Source: BMC Syst Biol. 2011 Jul 15;5:112. doi: 10.1186/1752-0509-5-112 (PMC3149001; doi:10.1186/1752-0509-5-112)
Supplement: Additional file 2 — Supplementary Table. Chemical reactions and related kinetic parameters used in this model. [file 1752-0509-5-112-S2.DOC]

# Supplementary Table S1

**Table S1 - List of chemical reactions and related kinetic parameters in model**.

The relevant references from which the parameters obtained are given. Some of the kinetics values used in this study are not necessary exactly the same as the values given in the cited references but were scaled and optimized in 10-fold ranges according to the performance and kinetics of current model (See Model Optimization and Validation under Materials and Methods section in main text for detailed description).

For those kinetics parameters that are not readily available, parameter values from their homologous partners were taken and were subsequently scaled and optimized in 10-fold ranges (denoted as “Estimated” in the Table).

“=” reversible reaction

“->” enzyme catalytic reaction

| **Reaction**  **Number** | **Chemical Reactions and Description** | **Kf**  **(uM.s)-1** | **Kb**  **(s)-1** | **Kcat (s)-1** | **References** |
| --- | --- | --- | --- | --- | --- |
|  | ***Activation of GPCR by thrombin*** |  |  |  |  |
| **1** | Thrombin + Pro_thrombinR (PAR-1) = Thrombin-Pro_thrombinR | 3 | 0.01 |  | [1] |
| **2** | Thrombin-Pro_thrombinR -> ThrombinR-active + Thrombin |  |  | 0.8 | [1] |
| **3** | ThrombinR-active + G12α_Gβγ_GDP = ThrombinR-active-G12α_Gβγ_GDP | 0.6 | 0.0006 |  | [1] |
| **4** | ThrombinR-active-G12α_Gβγ_GDP + GTP -> G12α_GTP+ Gβγ + GDP + ThrombinR |  |  | 0.1 | [1] |
| **5** | ThrombinR-active + Gqα_Gβγ_GDP= ThrombinR-active- Gqα_Gβγ_GDP | 0.68 | 0.0060 |  | [1] |
| **6** | ThrombinR-active- Gqα_Gβγ_GDP + GTP -> Gqα_GTP + Gβγ + GDP + ThrombinR |  |  | 0.08 | [1] |
| **7** | RGS + Gqα_GTP = RGS-Gqα_GTP | 0.8 | 1.0E-5 |  | [2] |
| **8** | RGS-Gqα_GTP -> RGS + Gqα_GDP |  |  | 0.88 | [2] |
| **9** | ThrombinR-active -> degradation |  |  | 0.002 | [2] |
| **10** | G12α_GTP -> G12α_GDP |  |  | 0.005 | [2] |
| **11** | Gqα_GTP ->Gqα_GDP |  |  | 0.0133 | [2] |
| **12** | Gβγ+ G12α_GDP = G12α_Gβγ_GDP | 0.01 | 0 |  | [2] |
| **13** | Gβγ+ Gqα_GDP = Gqα_Gβγ_GDP | 0.01 | 0 |  | [2] |
|  | ***Rho activation*** |  |  |  |  |
| **14** | RhoGEF + G12α_GTP = RhoGEF-G12α_GTP | 1.0 | 0.1 |  | [3] |
| **15** | RhoGEF-G12α_GTP = G12α_GDP + RhoGEF | 0.0117 | 0 |  | [3] |
| **16** | Rho_GDP + RhoGEF-G12α_GTP = RhoGEF-G12α_GTP-Rho_GDP | 7.6 | 0.0010 |  | [3] |
| **17** | RhoGEF-G12α_GTP-Rho_GDP -> Rho_GTP + RhoGEF-G12α_GTP |  |  | 0.07 | [3] |
| **18** | Rho_GTP + RhoGAP = Rho_GTP-RhoGAP | 8.0E-4 | 1.0E-4 |  | Estimated |
| **19** | Rho_GTP-RhoGAP-> Rho_GDP + RhoGAP |  |  | 0.08 | Estimated |
| **20** | Rho-GTP -> Rho-GDP |  |  | 3.6E-4 | [3] |
| **21** | Rho-GDP + RhoGDI = Rho-GDP-RhoGDI | 5.0 | 0.05 |  | [4] |
| **22** | pROCK -> degradation |  |  | 8.0E-4 | [3] |
|  | ***Ca^2+^ release*** |  |  |  |  |
| **23** | PLCβ + Ca^2+^ = PLCβ-Ca^2+^ | 0.8 | 0.001 |  | [5] |
| **24** | PLCβ-Ca^2+^ + PIP2 = PLCβ-Ca^2+^-PIP2 | 0.8 | 0.0001 |  | [5] |
| **25** | PLCβ_Ca^2+^-PIP2 -> IP3 + PLCβ_Ca^2+^ + DAG |  |  | 0.8 | [5] |
| **26** | PLCβ-Ca^2+^ + Gqα_GTP = PLCβ-Ca^2+^- Gqα_GTP | 1 | 1.0E-5 |  | [5] |
| **27** | PLCβ-Ca^2+^- Gqα_GTP + PIP2 = PLCβ-Ca^2+^- Gqα_GTP -PIP2 | 0.7 | 4.0E-4 |  | [5] |
| **28** | PLCβ-Ca^2+^- Gqα_GTP -PIP2 -> PLCβ-Ca^2+^- Gqα_GTP + IP3 + DAG |  |  | 0.68 | [5] |
| **29** | PLCβ + Gqα_GTP = PLCβ-Gqα_GTP | 1.5201 | 0.5 |  | Estimated |
| **30** | PLCβ-Gqα_GTP + Ca^2+^ = PLCβ-Ca^2+^- Gqα_GTP | 1.6 | 0.1 |  | [5] |
| **31** | PLCβ-Ca^2+^- Gqα_GTP = Gqα_GDP + PLCβ-Ca^2+^ | 0.0133 | 0 |  | [5] |
| **32** | DAG = PC | 0.15 | 0 |  | [5] |
| **33** | IP3 = Inositol | 0.01 | 0 |  | [5] |
| **34** | IP3R + IP3 + IP3 + IP3 = 3IP3-IP3R | 0.5 | 10 |  | [5] |
| **35** | ER.Ca2+_ store + IP3-IP3R = Ca2+-IP3-IP3R | 17.0 | 0.0010 |  | Estimated |
| **36** | Ca^2+^-IP3-IP3R -> IP3-IP3R + Ca^2+^ |  |  | 3 | [6] |
| **37** | Ca^2+^_ extleak + Ca^2+^_ ext = Ca^2+^_ ext1 | 0.0080 | 1.0E-4 |  | [6] |
| **38** | Ca^2+^_ ext1 -> Ca^2+^ + Ca^2+^_ extleak |  |  | 0.5 | [6] |
| **39** | ER.Ca^2+^_ store + Ca^2+^_ intleak = Ca^2+^_ int1 | 5.0 | 1.0E-4 |  | [6] |
| **40** | Ca^2+^_ int1 -> Ca^2+^_ intleak + Ca^2+^ |  |  | 0.5 | [6] |
| **41** | Ca^2+^_ pump + Ca^2+^ = Ca^2+^_ pump-Ca^2+^ | 6.0 | 1.0E-4 |  | [6] |
| **42** | Ca^2+^_ pump-Ca^2+^ -> Ca^2+^_ pump + Ca^2+^_ ext |  |  | 10.0 | [6] |
| **43** | 2Ca^2+^ + Ca^2+^_ trunsp = Ca^2+^_ trunsp-2Ca^2+^ | 10.0 | 0.3 |  | [6] |
| **44** | Ca^2+^_ trunsp-2Ca^2+^ + Ca^2+^_ trunsp = ER.Ca^2+^_ store | 5.0 | 1.0 |  | [6] |
| **45** | CaM + Ca^2+^ = Ca^2+^-CaM | 10.0 | 45.0 |  | [2, 7] |
| **46** | Ca^2+^-CaM + Ca^2+^ = CaM-2Ca^2+^ | 8.0 | 40.0 |  | Estimated |
| **47** | CaM-2Ca^2+^ + Ca^2+^ = CaM-3Ca^2+^ | 10.0 | 170.0 |  | [2, 7] |
| **48** | CaM-3Ca^2+^ + Ca^2+^ = CaM-4Ca^2+^ | 10.0 | 500 |  | [2, 7] |
| **49** | Ca^2+^-CaM + MLCK = MLCK-Ca^2+^-CaM | 0.03 | 0.08 |  | [2, 7] |
| **50** | CaM-2Ca^2+^ + MLCK = MLCK-2Ca^2+^-CaM | 0.04 | 0.15 |  | [2, 7] |
| **51** | CaM-3Ca^2+^ + MLCK = MLCK-3Ca^2+^-CaM | 1.0 | 7.0E-4 |  | [2, 7] |
| **52** | CaM-4Ca^2+^ + MLCK = MLCK-4Ca^2+^-CaM | 10.0 | 0.01 |  | [2, 7] |
|  | ***PKC activation*** |  |  |  |  |
| **53** | PKC + Ca^2+^ = PKC-Ca^2+^ | 0.3 | 0.01 |  | Estimated |
| **54** | PKC-Ca^2+^ + DAG = PKC-Ca^2+^-DAG | 0.3 | 0.0010 |  | Estimated |
| **55** | PKC-Ca^2+^-DAG = PKC_active | 1.78 | 0.01 |  | Estimated |
| **56** | PKC_active = degradation |  |  | 4.6E-4 | [8] |
| **57** | PKC_active + CPI-17 = PKC_active-CPI-17 | 3.2 | 0.01 |  | [9] |
| **58** | PKC_ active-CPI-17 -> PKC_ active + pCPI-17 |  |  | 1.68 | [9] |
| **59** | pCPI-17 = CPI-17 | 0.5 | 0.0010 |  | [9] |
| **60** | MYPT1_PPase + pCPI-17 = pCPI-17-MYPT1_PPasep | 7.2 | 0.62 |  | [9] |
| **61** | MYPT1_PPase + CPI-17 = CPI-17-MYPT1_PPase | 0.01 | 0.0010 |  | [9] |
| **62** | pCPI-17-MYPT1_PPasep = CPI-17-MYPT1_PPase | 0.0050 | 0.0010 |  | [9] |
|  | ***MLC activation*** |  |  |  |  |
| **63** | MLC + pROCK = pROCK-MLC | 2.02 | 0.0010 |  | [10] |
| **64** | pROCK-MLC -> pMLC + pROCK |  |  | 1.35 | [10] |
| **65** | pMLC + pROCK = pROCK-pMLC | 1.38 | 0.0010 |  | [10] |
| **66** | pROCK-pMLC -> pROCK + ppMLC |  |  | 0.07 | [10] |
| **67** | MLC + Rho-GTP-ROCK = Rho-GTP-ROCK-MLC | 0.05 | 1.0E-4 |  | [10] |
| **68** | Rho-GTP-ROCK-MLC -> pMLC + Rho-GTP-ROCK |  |  | 0.06 | [10] |
| **69** | pMLC + Rho-GTP-ROCK = Rho-GTP-ROCK-pMLC | 1.36 | 2.0E-4 |  | [10] |
| **70** | Rho-GTP-ROCK-pMLC -> ppMLC + Rho-GTP-ROCK |  |  | 1.6 | [10] |
| **71** | MLCK-Ca^2+^-CaM + MLC = MLCK-Ca^2+^-CaM -MLC | 0.8 | 1.0E-4 |  | [11] |
| **72** | MLCK-Ca^2+^-CaM-MLC -> MLCK-Ca^2+^-CaM + pMLC |  |  | 0.35 | [11] |
| **73** | MLCK-Ca^2+^-CaM + pMLC = MLCK-Ca^2+^-CaM-pMLC | 0.082 | 1.0E-4 |  | [11] |
| **74** | MLCK-Ca^2+^-CaM-pMLC -> ppMLC + MLCK-Ca^2+^-CaM |  |  | 0.25 | [11] |
| **75** | MLCK-2Ca^2+^-CaM + MLC = MLCK-2Ca^2+^-CaM-MLC | 0.5 | 0.0060 |  | [11] |
| **76** | MLCK-2Ca^2+^-CaM-MLC -> MLCK-2Ca^2+^-CaM + pMLC |  |  | 2.0 | [11] |
| **77** | MLCK-2Ca^2+^-CaM + pMLC = MLCK-2Ca^2+^-CaM-pMLC | 1.0 | 0.1 |  | [11] |
| **78** | MLCK-2Ca^2+^-CaM-pMLC -> MLCK-2Ca^2+^-CaM + ppMLC |  |  | 1.6 | [11] |
| **79** | MLCK-3Ca^2+^-CaM + MLC = MLCK-3Ca^2+^-CaM-MLC | 0.8 | 1.0E-5 |  | [11] |
| **80** | MLCK-3Ca^2+^-CaM-MLC -> pMLC + MLCK-3Ca^2+^-CaM |  |  | 0.3 | [11] |
| **81** | MLCK-3Ca^2+^-CaM + pMLC = MLCK-3Ca^2+^-CaM-pMLC | 0.4 | 1.0E-4 |  | [11] |
| **82** | MLCK-3Ca^2+^-CaM-pMLC -> MLCK-3Ca^2+^-CaM + ppMLC |  |  | 1.2 | [11] |
| **83** | MLCK-4Ca^2+^-CaM + MLC = MLCK-4Ca^2+^-CaM-MLC | 0.5 | 0.05 |  | [11] |
| **84** | MLCK-4Ca^2+^-CaM-MLC -> pMLC + MLCK-4Ca^2+^-CaM |  |  | 1.5 | [11] |
| **85** | MLCK-4Ca^2+^-CaM + pMLC = MLCK-4Ca^2+^-CaM-pMLC | 1.0 | 0.02 |  | [11] |
| **86** | MLCK-4Ca^2+^-CaM-pMLC -> ppMLC + MLCK-4Ca^2+^-CaM |  |  | 1.35 | [11] |
| **87** | MLCK + MLC = MLCK-MLC | 0.16 | 0.01 |  | Estimated |
| **88** | MLCK-MLC -> pMLC + MLCK |  |  | 0.6 | Estimated |
| **89** | MLCK + pMLC = MLCK-pMLC | 0.55 | 1.0E-5 |  | [11] |
| **90** | MLCK-pMLC -> ppMLC + MLCK |  |  | 0.2 | [11] |
| **91** | MYPT1_PPase + ppMLC = MYPT1_PPase-ppMLC | 0.15 | 1.0E-4 |  | [12] |
| **92** | MYPT1_PPase-ppMLC -> MYPT1_PPase + pMLC |  |  | 0.2 | [12] |
| **93** | MYPT1_PPase + pMLC = MYPT1_PPase-pMLC | 4.5 | 0.0010 |  | [12] |
| **94** | MYPT1_PPase-pMLC -> MLC + MYPT1_PPase |  |  | 1.68 | [12] |
| **95** | pMYPT1_PPase + ppMLC = pMYPT1_PPase-ppMLC | 0.08 | 1.0E-4 |  | [12] |
| **96** | pMYPT1_PPase-ppMLC -> pMLC + pMYPT1_PPase |  |  | 0.3 | [12] |
| **97** | pMYPT1_PPase + pMLC = pMYPT1_PPase-pMLC | 0.25 | 1.0E-4 |  | [12] |
| **98** | pMYPT1_PPase-pMLC -> MLC + pMYPT1_PPase |  |  | 0.7 | [12] |
| **99** | MYPT1_PPase + Rho-GTP-ROCK = Rho-GTP-ROCK-MYPT1_PPase | 1.0 | 0.0050 |  | Estimated |
| **100** | Rho-GTP-ROCK-MYPT1_PPase -> pMYPT1_PPase + Rho-GTP-ROCK |  |  | 2.5 | Estimated |
| **101** | MYPT1_PPase + pROCK = pROCK-MYPT1_PPase | 1.43 | 1.0E-4 |  | [11] |
| **102** | pROCK-MYPT1_PPase -> pMYPT1_PPase + pROCK |  |  | 0.72 | [11] |
| **103** | pMYPT1_PPase = MYPT1_PPase | 0.2 | 0 |  | [11] |
|  | ***ERK activation*** |  |  |  |  |
| **104** | VEGFR2 + VEGF = VEGF-VEGFR2 | 8.0 | 1.0E-4 |  | [13] |
| **105** | VEGF-VEGFR2 + VEGF-VEGFR2 = VEGF-VEGFR2-2 | 0.6 | 0.0010 |  | [13] |
| **106** | VEGF-VEGFR2-2 -> VEGF-pVEGFR2-2 |  |  | 2.01 | [13] |
| **107** | VEGF-pVEGFR2-2 + Shc = VEGF-pVEGFR2-2-Shc | 0.3 | 0.0060 |  | [14] |
| **108** | VEGF-pVEGFR2-2-Shc -> VEGF-pVEGFR2-2-pShc |  |  | 0.5838 | [14] |
| **109** | VEGF-pVEGFR2-2-pShc = pShc + VEGF-pVEGFR2-2 | 0.022 | 1.0E-4 |  | [14] |
| **110** | pShc + SHP = pShc-SHP | 0.05 | 1.0E-4 |  | [14] |
| **111** | pShc-SHP -> Shc + SHP |  |  | 0.28 | [14] |
| **112** | VEGF-pVEGFR2-2-pShc + Grb2 = VEGF-pVEGFR2-2-pShc-Grb2 | 3.0 | 0.1 |  | [15] |
| **113** | VEGF-pVEGFR2-2-pShc-Grb2 + Sos = VEGF-pVEGFR2-2-pShc-Grb2-SOS | 1.0 | 0.0214 |  | [15] |
| **114** | Grb2-Sos + VEGF-pVEGFR2-2-pShc = VEGF-pVEGFR2-2-pShc-Grb2-SOS | 1.0 | 0.045 |  | [15] |
| **115** | VEGF-pVEGFR2-2-pShc-Grb2-SOS + RasGDP = VEGF-pVEGFR2-2-pShc-Grb2-SOS-RasGDP | 9.2 | 0.0018 |  | [14] |
| **116** | VEGF-pVEGFR2-2-Grb2-SOS-RasGDP -> VEGF-pVEGFR2-2-Grb2-SOS + RasGTP |  |  | 10.56 | [14] |
| **117** | RasGTP = RasGDP | 3.0 | 1.0E-4 |  | [14] |
| **118** | RasGTP + RasGAP = RasGTP-RasGAP | 2.88 | 1.0E-4 |  | [14] |
| **119** | RasGTP-RasGAP -> RasGAP + RasGDP |  |  | 0.8 | [14] |
| **120** | VEGF-pVEGFR2-2 + Grb2 = VEGF-pVEGFR2-2-Grb2 | 1.67 | 0.02 |  | Estimated |
| **121** | VEGF-pVEGFR2-2-Grb2 + Sos = VEGF-pVEGFR2-2-Grb2-SOS | 6.0 | 0.03 |  | [14] |
| **122** | Grb2-Sos + VEGF-pVEGFR2-2 = VEGF-pVEGFR2-2-Grb2-SOS | 2.67 | 0.01 |  | [14] |
| **123** | VEGF-pVEGFR2-2-Grb2-SOS + RasGDP = VEGF-pVEGFR2-2-Grb2-SOS-RasGDP | 12.22 | 0.0016 |  | [14] |
| **124** | VEGF-pVEGFR2-2-Grb2-SOS-RasGDP -> VEGF-pVEGFR2-2-Grb2-SOS + RasGTP |  |  | 6.8 | [14] |
| **125** | Grb2 + Sos = Grb2-Sos | 0.1 | 0.0015 |  | [14] |
| **126** | RasGTP + Raf = RasGTP-Raf | 1.8 | 0.05 |  | [14] |
| **127** | RasGTP-Raf -> pRaf + RasGTP |  |  | 0.7624 | [14] |
| **128** | MEK + pRaf = pRaf-MEK | 4.0 | 0.01833 |  | [14] |
| **129** | pRaf-MEK -> pMEK + pRaf |  |  | 0.65 | [14] |
| **130** | pRaf + pMEK = pRaf-pMEK | 4.0 | 0.01833 |  | [14] |
| **131** | pRaf-pMEK -> pRaf + ppMEK |  |  | 2.9 | [14] |
| **132** | ppMEK + ERK = ppMEK-ERK | 0.86 | 0.0033 |  | [14] |
| **133** | ppMEK-ERK -> ppMEK + pERK |  |  | 2.88 | [14] |
| **134** | ppMEK + pERK = ppMEK-pERK | 3.98 | 0.0033 |  | [14] |
| **135** | ppMEK-pERK -> ppMEK + ppERK |  |  | 5.7 | [14] |
| **136** | ppERK -> degradation |  |  | 0.0025 | [14] |
| **137** | pRaf + Pase = pRaf-Pase | 6.0 | 0.2 |  | [16] |
| **138** | pRaf-Pase -> Pase + Raf |  |  | 1.0 | [16] |
| **139** | ppMEK + PP2A = ppMEK-PP2A | 3.0 | 0.8 |  | [16] |
| **140** | ppMEK-PP2A -> pMEK + PP2A |  |  | 0.1 | [16] |
| **141** | pMEK + PP2A = pMEK-PP2A | 3.0 | 0.8 |  | [16] |
| **142** | pMEK-PP2A -> PP2A + MEK |  |  | 0.1 | [16] |
| **143** | MKP3 + ppERK = ppERK-MKP3 | 0.8 | 0.0060 |  | [16] |
| **144** | ppERK-MKP3 -> pERK + MKP3 |  |  | 0.27 | [16] |
| **145** | pERK + MKP3 = pERK-MKP3 | 4.25 | 5.0E-4 |  | [16] |
| **146** | pERK-MKP3 -> MKP3 + ERK |  |  | 0.3 | [16] |
| **147** | ppERK + VEGF-pVEGFR2-2-pShc-Grb2-SOS = ppERK-VEGF-pVEGFR2-2-pShc-Grb2-SOS | 20.898 | 1.0 |  | [14] |
| **148** | ppERK-VEGF-pVEGFR2-2-pShc-Grb2-SOS -> ppERK + VEGF-pVEGFR2-2 + pShc + pSOS |  |  | 0.0426 | [14] |
| **149** | ppERK + VEGF-pVEGFR2-2-Grb2-SOS = ppERK-VEGF-pVEGFR2-2-Grb2-SOS | 20.898 | 1.0 |  | [14] |
| **150** | ppERK-VEGF-pVEGFR2-2-Grb2-SOS -> ppERK + VEGF-pVEGFR2-2 + Grb2 + pSOS |  |  | 0.0426 | [14] |
| **151** | pSOS -> Sos |  |  | 0.02 | [17] |
| **152** | ppERK + MLCK = ppERK-MLCK | 0.44 | 6.0E-4 |  | [18] |
| **153** | ppERK-MLCK -> pMLCK + ppERK |  |  | 0.943 | [18] |
| **154** | ppERK + pMLCK = ppERK-pMLCK | 0.05 | 1.0E-5 |  | [18] |
| **155** | ppERK-MLCK -> pMLCK + ppERK |  |  | 0.0030 | [18] |
| **156** | ppMLCK + MLC = ppMLCK-MLC | 0.08 | 1.0E-5 |  | [18] |
| **157** | ppMLCK-MLC -> ppMLCK + pMLC |  |  | 0.97 | [18] |
| **158** | ppMLCK + pMLC = ppMLCK-pMLC | 2.96 | 1.0E-4 |  | [18] |
| **159** | ppMLCK-pMLC -> ppMLCK + ppMLC |  |  | 0.46 | [18] |
| **160** | MLC + ppERK-pMLCK = ppERK-pMLCK-MLC | 0.0080 | 1.0E-5 |  | [18] |
| **161** | ppERK-pMLCK-MLC -> ppERK-pMLCK + pMLC |  |  | 0.4 | [18] |
| **162** | ppERK-pMLCK + pMLC = ppERK-pMLCK-pMLC | 15.64 | 0.0010 |  | [18] |
| **163** | ppERK-pMLCK-pMLC -> ppMLC + ppERK-pMLCK |  |  | 4.8 | [18] |
| **164** | ppMLCK -> degradation |  |  | 8.0E-4 | [18] |
|  | ***Activation of Ca^2+^ and PKC by VEGF*** |  |  |  |  |
| **165** | PLC_γ+ Ca^2+^ = PLC_γ-Ca^2+^ | 0.8 | 0.0010 |  | [5] |
| **166** | PIP2 + PLC_γ-Ca^2+^ = PLC_γ-Ca^2+^-PIP2 | 0.78 | 1.0E-4 |  | [5] |
| **167** | PLC_γ-Ca^2+^-PIP2 -> IP3 + DAG + PLC_gamma-Ca^2+^ |  |  | 0.8 | [5] |
| **168** | PLC_γ-Ca^2+^ + VEGF-pVEGFR2-2 = PLC_γ-Ca^2+^ -VEGF-pVEGFR2-2 | 1.5 | 0.01 |  | [5] |
| **169** | Ca^2+^ + VEGF-pVEGFR2-2- PLC_γ= VEGF-pVEGFR2-2- PLC_γ-Ca^2+^ |  |  |  | [5] |
| **170** | PIP2 + PLC_γ-Ca^2+^ -VEGF-pVEGFR2-2 =  PLC_γ-Ca^2+^ -VEGF-pVEGFR2-2-PIP2 | 0.78 | 4.0E-4 |  | [5] |
| **171** | VEGF-pVEGFR2-2-PLC_gamma-Ca^2+^-PIP2 -> VEGF-pVEGFR2-2-PLC_gamma-Ca^2+^ + IP3 + DAG |  |  | 0.5 | [5] |
| **172** | PLC_γ+ VEGF-pVEGFR2-2 = VEGF-pVEGFR2-2- PLC_γ | 1.52 | 0.1 |  | Estimated |
|  | ***NO-PKG-activation*** |  |  |  |  |
| **173** | VEGF-pVEGFR2-2 + eNOS = VEGF-pVEGFR2-2-eNOS | 55.0 | 1.0E-4 |  | [19] |
| **174** | VEGF-pVEGFR2-2-eNOS -> eNOS-active + VEGF-pVEGFR2-2 |  |  | 0.25 | [19] |
| **175** | H1R-active + eNOS = H1R-active-eNOS | 55.0 | 1.0E-4 |  | [19] |
| **176** | H1R-active-eNOS -> H1R-active + eNOS-active |  |  | 0.6 | [19] |
| **177** | eNOS-active + L-Arg = eNOS-active-L-Arg | 20.0 | 0.01 |  | Estimated |
| **178** | eNOS-active-L-Arg -> L-Cit + eNOS-active + NO |  |  | 1.2 | [19] |
| **179** | NO + GC = NO-GC | 10.0 | 0.1 |  | [19] |
| **180** | NO-GC + GTP = NO-GC-GTP | 2.11 | 0.05 |  | [19] |
| **181** | NO-GC-GTP -> NO-GC + cGMP |  |  | 0.8 | [19] |
| **182** | cGMP + PKG = cGMP-PKG | 0.68 | 0.0010 |  | [19] |
| **183** | cGMP-PKG -> PKG-active + cGMP |  |  | 0.06 | [19] |
| **184** | PKG-active + Raf = PKG-Raf | 0.22 | 0.0010 |  | Estimated |
| **185** | PKG-Raf -> PKG-active + pRaf |  |  | 0.21 | [20] |
| **186** | NO -> NO1 |  |  | 0.15 | [19] |
| **187** | NO -> NO2 |  |  | 0.25 | [19] |
| **188** | NO -> NO3 |  |  | 0.25 | [19] |
| **189** | PKG-active -> degradation |  |  | 0.0010 | [20] |
|  | ***Activation of Gqα and Ca^2+^ by histamine*** |  |  |  |  |
| **190** | Histamine + H1R = Histamine-H1R | 3 | 0.01 |  | [21] |
| **191** | Histamine-H1R -> H1R-active + Histamine |  |  | 0.8 | [21] |
| **192** | H1R -> degradation |  |  | 0.0022 | [21] |
| **193** | H1R-active -> degradation |  |  | 0.0133 | [21] |
| **194** | H1R-active + Gqα_Gβγ_GDP = Gqα_Gβγ_GDP -H1R-active | 0.68 | 0.0006 |  | [21] |
| **195** | GTP + Gqα_Gβγ_GDP -H1R-active -> GDP + Gqα_GTP + Gβγ_GDP |  |  | 0.08 | [21] |
| **196** | Rho-GTP + ROCK-fold = ROCK-open + Rho-GTP | 25 | 0 |  | [22] |
| **197** | ROCK-open + Rho-GTP = Rho-GTP-ROCK-open | 0.05 | 0.0005 |  | [22] |
| **198** | Rho-GTP-ROCK-open -> pROCK + Rho-GTP |  |  | 0.085 | [23] |
| **199** | PKG-active + ROCK-open = PKG-ROCK-open | 0.35 | 0.0001 |  | [23] |
| **200** | PKG-ROCK-open -> pROCK + PKG |  |  | 1.85 | [23] |
| **Initial Concentration** | | | | | |
| **ID** | **Component** | **Concentration** | | **References**  **(PubMed ID)** | |
| **1** | Pro_thrombinR (PAR-1) | 0.05 | | [24] | |
| **2** | RGS | 0.2 | | [24] | |
| **3** | GTP | 50 | | [24] | |
| **4** | GDP | 5 | | [24] | |
| **5** | IP3R | 0.33 | | Estimated | |
| **6** | RhoGEF | 0.25 | | Estimated | |
| **7** | RhoGAP | 0.15 | | [17] | |
| **8** | PIP2 | 10.0 | | [25] | |
| **9** | Rho-GDP | 0.1 | | [17] | |
| **10** | MLCK | 0.69 | | [17] | |
| **11** | PKC | 0.2 | | [26] | |
| **12** | CPI-17 | 0.08 | | [26] | |
| **13** | MLC | 4.2 | | [27] | |
| **14** | pMLC | 0.6 | | [27] | |
| **15** | Ca^2+^ | 0.0083 | | [25] | |
| **16** | CaM | 20 | | [25] | |
| **17** | PLCβ | 0.57 | | [25] | |
| **18** | Ca^2+^_ trunsp | 20.0 | | Estimated | |
| **19** | Ca^2+^_ pump | 0.08 | | Estimated | |
| **20** | ppMLC | 0.8 | | [27] | |
| **21** | MYPT1_PPase | 0.4 | | [26] | |
| **22** | pMYPT1_PPase | 0.01 | | Estimated | |
| **23** | G12α_Gβγ_GDP | 0.4 | | [24] | |
| **24** | Gqα_Gβγ_GDP | 0.5 | | [24] | |
| **25** | Ca^2+^_ extleak | 4.0 | | Estimated | |
| **26** | Ca^2+^_ intleak | 0.8 | | Estimated | |
| **27** | RhoGDI | 0.05 | | [3] | |
| **28** | ROCK | 0.16 | | [3] | |
| **29** | VEGF | 0.02 | | Estimated | |
| **30** | VEGFR2 | 0.02 | | Estimated | |
| **31** | SHP | 0.25 | | [17] | |
| **32** | Grb2 | 0.15 | | [17] | |
| **33** | Sos | 0.12 | | [17] | |
| **34** | RasGDP | 0.5 | | [17] | |
| **35** | Shc | 0.5 | | [17] | |
| **36** | Raf | 0.5 | | [17] | |
| **37** | MEK | 0.05 | | [17] | |
| **38** | ERK | 0.05 | | [17] | |
| **39** | Pase | 0.5 | | [17] | |
| **40** | PP2A | 0.02 | | [17] | |
| **41** | MKP3 | 0.01 | | [17] | |
| **42** | RasGAP | 0.8 | | [17] | |
| **43** | PLC_γ | 0.57 | | [25] | |
| **44** | eNOS | 0.33 | | [19] | |
| **45** | GC | 0.05 | | [19] | |
| **46** | PKG | 0.015 | | [19] | |
| **47** | L-Arg | 0.55 | | [19] | |
| **48** | H1R | 0.35 | | [21] | |

Supplementary References

1. Parry, M.A., et al., *Cleavage of the thrombin receptor: identification of potential activators and inactivators.* Biochem J, 1996. **320 ( Pt 1)**: p. 335-41.

2. Maeda, A., et al., *Ca2+ -independent phospholipase A2-dependent sustained Rho-kinase activation exhibits all-or-none response.* Genes Cells, 2006. **11**(9): p. 1071-83.

3. Kozasa, T., et al., *p115 RhoGEF, a GTPase activating protein for Galpha12 and Galpha13.* Science, 1998. **280**(5372): p. 2109-11.

4. Fujita, H., et al., *Molecular decipherment of Rho effector pathways regulating tight-junction permeability.* Biochem J, 2000. **346 Pt 3**: p. 617-22.

5. Lukas, T.J., *A signal transduction pathway model prototype I: From agonist to cellular endpoint.* Biophys J, 2004. **87**(3): p. 1406-16.

6. Meyer, T. and L. Stryer, *Molecular model for receptor-stimulated calcium spiking.* Proc Natl Acad Sci U S A, 1988. **85**(14): p. 5051-5.

7. Fajmut, A., A. Dobovisek, and M. Brumen, *Mathematical modeling of the relation between myosin phosphorylation and stress development in smooth muscles.* J Chem Inf Model, 2005. **45**(6): p. 1610-5.

8. Newton, A.C., *Protein kinase C: structure, function, and regulation.* J Biol Chem, 1995. **270**(48): p. 28495-8.

9. Eto, M., et al., *Molecular cloning of a novel phosphorylation-dependent inhibitory protein of protein phosphatase-1 (CPI17) in smooth muscle: its specific localization in smooth muscle.* FEBS Lett, 1997. **410**(2-3): p. 356-60.

10. Anderson, S., et al., *Rho-mediated assembly of stress fibers is differentially regulated in corneal fibroblasts and myofibroblasts.* Exp Cell Res, 2004. **298**(2): p. 574-83.

11. Lukas, T.J., *A signal transduction pathway model prototype II: Application to Ca2+-calmodulin signaling and myosin light chain phosphorylation.* Biophys J, 2004. **87**(3): p. 1417-25.

12. Feng, J., et al., *Inhibitory phosphorylation site for Rho-associated kinase on smooth muscle myosin phosphatase.* J Biol Chem, 1999. **274**(52): p. 37385-90.

13. Matsumoto, T. and H. Mugishima, *Signal transduction via vascular endothelial growth factor (VEGF) receptors and their roles in atherogenesis.* J Atheroscler Thromb, 2006. **13**(3): p. 130-5.

14. Yamada, S., T. Taketomi, and A. Yoshimura, *Model analysis of difference between EGF pathway and FGF pathway.* Biochem Biophys Res Commun, 2004. **314**(4): p. 1113-20.

15. Kholodenko, B.N., et al., *Quantification of short term signaling by the epidermal growth factor receptor.* J Biol Chem, 1999. **274**(42): p. 30169-81.

16. Schoeberl, B., et al., *Computational modeling of the dynamics of the MAP kinase cascade activated by surface and internalized EGF receptors.* Nat Biotechnol, 2002. **20**(4): p. 370-5.

17. Sasagawa, S., et al., *Prediction and validation of the distinct dynamics of transient and sustained ERK activation.* Nat Cell Biol, 2005. **7**(4): p. 365-73.

18. Klemke, R.L., et al., *Regulation of cell motility by mitogen-activated protein kinase.* J Cell Biol, 1997. **137**(2): p. 481-92.

19. Roy, B. and J. Garthwaite, *Nitric oxide activation of guanylyl cyclase in cells revisited.* Proc Natl Acad Sci U S A, 2006. **103**(32): p. 12185-90.

20. Hood, J. and H.J. Granger, *Protein kinase G mediates vascular endothelial growth factor-induced Raf-1 activation and proliferation in human endothelial cells.* J Biol Chem, 1998. **273**(36): p. 23504-8.

21. van Nieuw Amerongen, G.P., et al., *Transient and prolonged increase in endothelial permeability induced by histamine and thrombin: role of protein kinases, calcium, and RhoA.* Circ Res, 1998. **83**(11): p. 1115-23.

22. Riento, K. and A.J. Ridley, *Rocks: multifunctional kinases in cell behaviour.* Nat Rev Mol Cell Biol, 2003. **4**(6): p. 446-56.

23. Sunico, C.R., et al., *Nitric oxide induces pathological synapse loss by a protein kinase G-, Rho kinase-dependent mechanism preceded by myosin light chain phosphorylation.* J Neurosci, 2010. **30**(3): p. 973-84.

24. Tiruppathi, C., et al., *Thrombin receptor 14-amino acid peptide binds to endothelial cells and stimulates calcium transients.* Am J Physiol, 1992. **263**(5 Pt 1): p. L595-601.

25. Bhalla, U.S. and R. Iyengar, *Emergent properties of networks of biological signaling pathways.* Science, 1999. **283**(5400): p. 381-7.

26. MacDonald, J.A., et al., *Dual Ser and Thr phosphorylation of CPI-17, an inhibitor of myosin phosphatase, by MYPT-associated kinase.* FEBS Lett, 2001. **493**(2-3): p. 91-4.

27. Goeckeler, Z.M. and R.B. Wysolmerski, *Myosin light chain kinase-regulated endothelial cell contraction: the relationship between isometric tension, actin polymerization, and myosin phosphorylation.* J Cell Biol, 1995. **130**(3): p. 613-27.
