# Supplementary material for: An Integrated Mathematical Model of Thrombin-, Histamine-and VEGF-Mediated Signalling in Endothelial Permeability
Source: BMC Syst Biol. 2011 Jul 15;5:112. doi: 10.1186/1752-0509-5-112 (PMC3149001; doi:10.1186/1752-0509-5-112)
Supplement: Additional file 3 — Supplementary Model Description. Detail description of signaling cascades used in this model. [file 1752-0509-5-112-S3.DOC]

# Supplementary Material 1

# 1 Supplementary Model Description

A detail description of signaling model used in this study is given below. The equation numbers in the text refer to the equation number given in Supplementary Table S1.

**Thrombin mediated Rho GTPase activation**

**Rho GTPase activation:**

The Rho GTPase cycle is tightly regulated by three groups of proteins. Guanine nucleotide exchange factors (GEFs) promote the exchange of GDP for GTP to activate the GTPase, GTPase-activating proteins (GAPs) negatively regulate the switch by enhancing its intrinsic GTPase activity and guanine nucleotide dissociation inhibitors (GDIs) are thought to block the GTPase cycle by sequestering and solubilizing the GDP-bound form [1-3]. Extracellular signals could regulate the switch by modifying any of these proteins, but so far at least, they appear to act predominantly through GEFs. Once activated, Rho GTPases interact with cellular target proteins (effectors) to generate a downstream response (Figure S15 ) [4].

**Figure 1: The Rho GTPase cycle.**

Rho GTPases cycle between an inactive GDP-bound form and an active GTP-bound form. The cycle is tightly regulated mainly by guanine exchange factors (GEFs), GTPase activating proteins (GAPs) and guanine dissociation inhibitors (GDIs). In their active form, Rho GTPases can bind to effector molecules such as kinases and scaffold proteins.

**
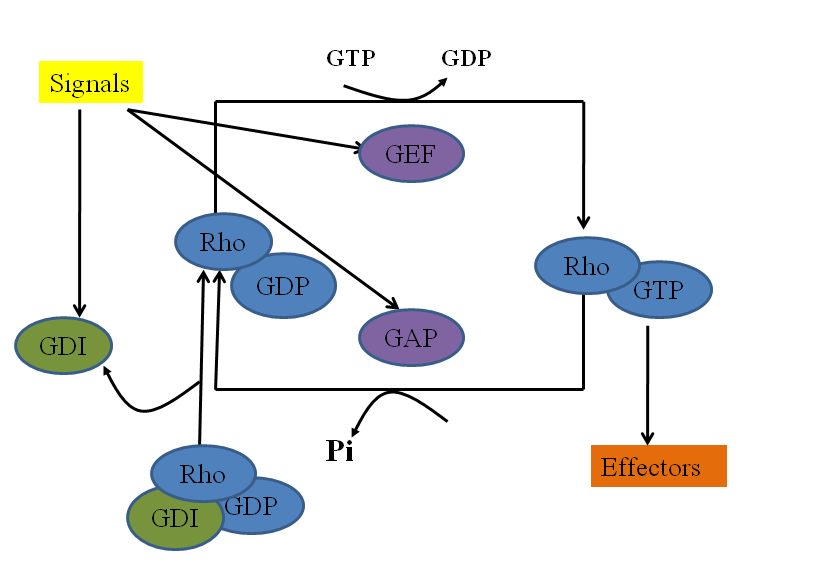
**

**Role of MAP Kinase in Cell Migration**

VEGF can activate ERK-1/2 through the typical signaling of Raf-MEK-ERK pathway [5]. For the subsequent signaling of MLC activation, there are accumulating evidences that ERK-MLCK-meditated cytoskeletal responses contribute to VEGF-elicited microvascular hyperpermeability. Shoemaker et al. has examined that MLCK contains multiple MAP kinase consensus phosphorylation sites (P-x-S[6]-P) and it can be directly phosphorylated by MAP kinase [7]. Evidence presented in by Richard’s experiment demonstrates that MLCK, a key regulator of cell motility and contraction, is a substrate for MAP kinase [8].

**Thrombin, VEGF and Histamine mediated Ca^2+^ release, PKC activation**

1. **Ca^2+^ release:**

In endothelial cells, binding to GPCRs by agonists causes Gαq to switch from a GDP-bound to a GTP-bound state, allowing the release of Gαq from the Gβγ dimer. The GTP bound Gαq subunit subsequently activates phosphoinositide phospholipase PLC-β, which then hydrolyses the lipid precursor phosphatidylinositol-4,5-bisphosphate (PIP2) to yield IP3 and diacylglycerol. IP3 receptors constitute the most clearly identified Ca^2+^ channels that pump Ca^2+^ from the ER. Most cells have at least one form of IP3 receptor, and many express all three. Structurally, the IP3 receptor channels are tetramers composed of four subunits, IP3-mediated Ca^2+^ release responses are co-operative, indicating that several and perhaps all subunits are required to bind IP3 for the channel to open [9] (Figure S16). A characteristic feature of IP3 receptors is that they are regulated by both IP3 and Ca^2+^.

**Figure 2: GPCR activation and Ca^2+^ relaease.**

External stimulus activates a G-Protein-Coupled Receptor (GPCR), which activates a stimulating G-protein. The G-protein activates phospholipase C (PLC), which cleaves phosphoinositol-4,5-bisphosphate (PIP2) into 1,2-diacylglycerol and inositol-1,4,5-trisphosphate (IP3). The IP3 interacts with a calcium channel in the endoplasmic reticulum (ER), releasing Ca^2+^ into the cytoplasm. The increase in Ca^2+^ levels activates PKC, which translocates to the membrane, anchoring to diacylglycerol (DAG) and phosphatidylserine [10] (From Promega signal transduction, Source: [Signal Transduction Resource](http://www.promega.com/guides/sigtrans_guide/), [www.promega.com](http://www.promega.com/)).


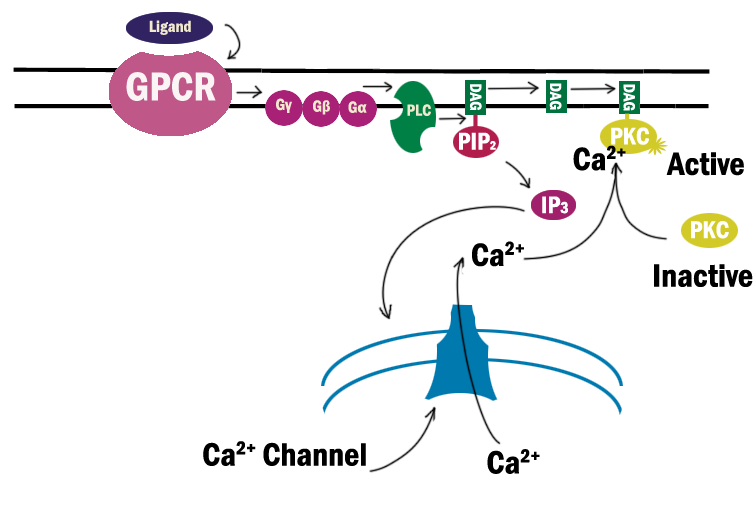


1. **PKC activation:**

PKC activation occurs when plasma membrane receptors coupled to phospholipase C are activated, releasing diacylglycerol (Supplementary Figure S4). The conventional isoforms, α, βI, βII, and γ, are activated by phosphatidylserine, diacylglycerol and Ca^2+^. The unconventional isoforms, δ, ε, η, and θ, require phosphatidylserine and diacylglycerol but do not require Ca^2+^. The ζ and λ isoforms are called atypical and require only phosphatidylserine for activation. The G-protein activates phospholipase C (PLC), which cleaves phosphoinositol-4,5-bisphosphate (PIP2) into 1,2-diacylglycerol and inositol-1,4,5-trisphosphate (IP3). The IP3 interacts with a calcium channel in the endoplasmic reticulum (ER), releasing Ca^2+^ into the cytoplasm. The increase in Ca^2+^ levels activates PKC, which translocates to the membrane, anchoring to diacylglycerol (DAG) and phosphatidylserine.

1. **MLC activation:**

Phosphorylation of regulatory light chain (MLC) of myosin II plays a critical role in controlling actomyosin contractility in both smooth muscle and nonmuscle cells [11-13]. MLC phosphorylation is regulated by the balance of two enzymatic activities, i.e., Myosin light chain kinase (MLCK) and myosin phosphatase (MYCP). MLCK is regulated by Ca^2+^ /calmodulin and is believed to be a major kinase in both smooth muscle and nonmuscle cells. In addition, Rho-kinase can directly phosphorylate MLC in vitro [14]. MYCP is a holoenzyme composed of three subunits: a catalytic subunit of 38 kDa that was identified as protein phosphatase 1 (PP1) catalytic subunit δ-isoform (PP1Cδ) [15] and two noncatalytic subunits of 21 and 110–130 kDa [16, 17]. The larger one, called myosin phosphatase targeting subunit 1 (MYPT1), binds to the catalytic subunit and targets it to MLC, providing substrate specificity [18]. Rho-kinase (RhoK) and protein kinase C (PKC) have been proposed to mediate the inhibition of smooth muscle MYCP, leading to increased MLC phosphorylation in response to various agonists. Phosphorylation of the MYPT1 regulatory site (Thr695 in chicken MYPT1) by RhoK induces inhibition of MYCP activity [19]. A number of experimental facts suggest that CPI-17 (for PKC-potentiated inhibitory protein of 17 kDa) is involved in PKC-dependent inhibition of MYCP. CPI-17 is a soluble globular protein described as a specific inhibitor for MYCP [20].

**2 Supplementary Methods: Estimation of kinetic parameters**

We estimated the values of unknown parameters in the model when some parameters cannot be determined from direct experiments or the literatures. Unknown or only roughly known parameters were estimated by minimizing the discrepancy between the experiment data and model simulation. These parameters were determined by using the trust regions algorithm [21] to fit the simulated to the experimental data of RAS, ERK, MYPT and CPI-17 activation curves [22-24]. The level of fitting is based on the least-squares method and the fitting process proceeds in iterations until the R-square value is >0.6.

The procedure can be summarized as follows:

1. Import target experiment data
2. Simulate the model with the rough parameter values
3. Compute R-Square value for the simulation and experiment data before parameter estimation
4. Set up the parameters to estimate and the state to match.
5. Use the current values of parameters in the model as the starting point for optimization
6. Simulating the model with the new estimate parameters and computing R-Square value for comparison.
7. Plot the results

For example, we estimated parameters by fit our simulation results to experiment data of Activated Ras [23]. The experiment data, simulation results before and after parameter estimation were shown as Figure S14. The fitness has been improved with R-Square value from 0.4508 to 0.6088.

**Supplementary References**

1. Moon SY, Zheng Y: **Rho GTPase-activating proteins in cell regulation.** *Trends Cell Biol* 2003, **13:**13-22.

2. Schmidt A, Hall A: **Guanine nucleotide exchange factors for Rho GTPases: turning on the switch.** *Genes Dev* 2002, **16:**1587-1609.

3. Zheng Y: **Dbl family guanine nucleotide exchange factors.** *Trends Biochem Sci* 2001, **26:**724-732.

4. Fujita H, Katoh H, Hasegawa H, Yasui H, Aoki J, Yamaguchi Y, Negishi M: **Molecular decipherment of Rho effector pathways regulating tight-junction permeability.** *Biochem J* 2000, **346 Pt 3:**617-622.

5. Cobb MH: **MAP kinase pathways.** *Prog Biophys Mol Biol* 1999, **71:**479-500.

6. Aixinjueluo W, Furukawa K, Zhang Q, Hamamura K, Tokuda N, Yoshida S, Ueda R: **Mechanisms for the apoptosis of small cell lung cancer cells induced by anti-GD2 monoclonal antibodies: roles of anoikis.** *J Biol Chem* 2005, **280:**29828-29836.

7. Shoemaker MO, Lau W, Shattuck RL, Kwiatkowski AP, Matrisian PE, Guerra-Santos L, Wilson E, Lukas TJ, Van Eldik LJ, Watterson DM: **Use of DNA sequence and mutant analyses and antisense oligodeoxynucleotides to examine the molecular basis of nonmuscle myosin light chain kinase autoinhibition, calmodulin recognition, and activity.** *J Cell Biol* 1990, **111:**1107-1125.

8. Klemke RL, Cai S, Giannini AL, Gallagher PJ, de Lanerolle P, Cheresh DA: **Regulation of cell motility by mitogen-activated protein kinase.** *J Cell Biol* 1997, **137:**481-492.

9. Meyer T, Stryer L: **Molecular model for receptor-stimulated calcium spiking.** *Proc Natl Acad Sci U S A* 1988, **85:**5051-5055.

10. Cheng Z, Garvin D, Paguio A, Stecha P, Wood K, Fan F: **Luciferase Reporter Assay System for Deciphering GPCR Pathways.** *Curr Chem Genomics* 2010, **4:**84-91.

11. Kamm KE, Stull JT: **The function of myosin and myosin light chain kinase phosphorylation in smooth muscle.** *Annu Rev Pharmacol Toxicol* 1985, **25:**593-620.

12. Moussavi RS, Kelley CA, Adelstein RS: **Phosphorylation of vertebrate nonmuscle and smooth muscle myosin heavy chains and light chains.** *Mol Cell Biochem* 1993, **127-128:**219-227.

13. Somlyo AP, Somlyo AV: **Signal transduction and regulation in smooth muscle.** *Nature* 1994, **372:**231-236.

14. Amano M, Ito M, Kimura K, Fukata Y, Chihara K, Nakano T, Matsuura Y, Kaibuchi K: **Phosphorylation and activation of myosin by Rho-associated kinase (Rho-kinase).** *J Biol Chem* 1996, **271:**20246-20249.

15. Hartshorne DJ, Ito M, Erdodi F: **Myosin light chain phosphatase: subunit composition, interactions and regulation.** *J Muscle Res Cell Motil* 1998, **19:**325-341.

16. Alessi D, MacDougall LK, Sola MM, Ikebe M, Cohen P: **The control of protein phosphatase-1 by targetting subunits. The major myosin phosphatase in avian smooth muscle is a novel form of protein phosphatase-1.** *Eur J Biochem* 1992, **210:**1023-1035.

17. Shirazi A, Iizuka K, Fadden P, Mosse C, Somlyo AP, Somlyo AV, Haystead TA: **Purification and characterization of the mammalian myosin light chain phosphatase holoenzyme. The differential effects of the holoenzyme and its subunits on smooth muscle.** *J Biol Chem* 1994, **269:**31598-31606.

18. Johnson D, Cohen P, Chen MX, Chen YH, Cohen PT: **Identification of the regions on the M110 subunit of protein phosphatase 1M that interact with the M21 subunit and with myosin.** *Eur J Biochem* 1997, **244:**931-939.

19. Kimura K, Ito M, Amano M, Chihara K, Fukata Y, Nakafuku M, Yamamori B, Feng J, Nakano T, Okawa K, et al: **Regulation of myosin phosphatase by Rho and Rho-associated kinase (Rho-kinase).** *Science* 1996, **273:**245-248.

20. Eto M, Ohmori T, Suzuki M, Furuya K, Morita F: **A novel protein phosphatase-1 inhibitory protein potentiated by protein kinase C. Isolation from porcine aorta media and characterization.** *J Biochem* 1995, **118:**1104-1107.

21. Coleman TF, Li YY: **An interior trust region approach for nonlinear minimization subject to bounds.** *Siam J Optimiz* 1996, **6:**418-445.

22. Kawano Y, Fukata Y, Oshiro N, Amano M, Nakamura T, Ito M, Matsumura F, Inagaki M, Kaibuchi K: **Phosphorylation of myosin-binding subunit (MBS) of myosin phosphatase by Rho-kinase in vivo.** *J Cell Biol* 1999, **147:**1023-1038.

23. Sasagawa S, Ozaki Y, Fujita K, Kuroda S: **Prediction and validation of the distinct dynamics of transient and sustained ERK activation.** *Nat Cell Biol* 2005, **7:**365-373.

24. Dimopoulos GJ, Semba S, Kitazawa K, Eto M, Kitazawa T: **Ca2+-dependent rapid Ca2+ sensitization of contraction in arterial smooth muscle.** *Circ Res* 2007, **100:**121-129.
